# The developing leaf of the wild grass *Brachypodium distachyon* at single-cell resolution

**DOI:** 10.1093/plcell/koag172

**Published:** 2026-06-10

**Authors:** Lea S Berg, Paola Ruiz Duarte, Inés Hidalgo Prados, Nathan T Lacombe, Rashmi Tandon, Isaia Vardanega, Jan E Maika, Roxane P Spiegelhalder, Ambuj Gore, Heike Lindner, Rüdiger Simon, Michael T Raissig

**Affiliations:** Institute of Plant Sciences, University of Bern, Bern 3013, Switzerland; Institute of Plant Sciences, University of Bern, Bern 3013, Switzerland; Centre for Organismal Studies, Heidelberg University, Heidelberg 69120, Germany; Institute of Plant Sciences, University of Bern, Bern 3013, Switzerland; Centre for Organismal Studies, Heidelberg University, Heidelberg 69120, Germany; Institute of Developmental Genetics, Heinrich-Heine University, Düsseldorf, Germany; Institute of Developmental Genetics, Heinrich-Heine University, Düsseldorf, Germany; Institute of Plant Sciences, University of Bern, Bern 3013, Switzerland; Centre for Organismal Studies, Heidelberg University, Heidelberg 69120, Germany; Institute of Plant Sciences, University of Bern, Bern 3013, Switzerland; Oeschger Centre for Climate Change Research, University of Bern, Bern 3012, Switzerland; Institute of Developmental Genetics, Heinrich-Heine University, Düsseldorf, Germany; Institute of Plant Sciences, University of Bern, Bern 3013, Switzerland; Oeschger Centre for Climate Change Research, University of Bern, Bern 3012, Switzerland

## Abstract

Leaves are the plant's main photosynthetic organs and drive Earth's primary production. Grasses form longitudinal leaves with parallel venation and graminoid stomata. Yet, how distinct leaf tissues coordinately develop to build functional grass leaf anatomy is not well understood. Here, we decoded the developing grass leaf from vegetative meristems to mature tissues using single-cell RNA-sequencing in the wild grass *Brachypodium distachyon*. In-depth analysis of epidermal clusters and multiplexed whole-mount RNA-fluorescence in situ hybridization resolved most epidermal lineages and confirmed them *in planta*. Gene regulatory network analysis distinguished the targetomes of the 2 co-expressed, yet functionally divergent stomatal transcription factors *BdMUTE* and *BdFAMA*. Finally, we used our dataset to identify and functionally describe the role of the transcription factor gene *BdGRAS32* in inhibiting cell division and a stomata-specific role for the putative cell wall-modifying enzyme *BdPME53-like*. Our single-cell grass leaf atlas enables the dissection of developmental processes that make the grass leaf, which sustains global food production.

## Teaser

A single-cell transcriptomic atlas resolved the developmental transitions from shoot apex to mature leaf tissues in the model grass *Brachypodium distachyon*.

## Introduction

More than 40% of the Earth's surface is dominated by grasses (Beer et al. 2010), and the 3 cereal crops maize, rice, and wheat directly produce more than half of the calories consumed by humans (FAO Statistical Pocketbook 2015). Thus, the grass leaf is the photosynthetic powerhouse that fuels ecosystems and our civilization alike. The longitudinal sheathing leaf of grasses and other monocots is an evolutionary innovation, where the leaf sheath protects the shoot apical meristem and developing leaves (Richardson et al. 2021). The parallel venation provides additive water transport capacity (Nobel 2020), and morphological improvements to the graminoid stomata (Spiegelhalder and Raissig 2021; Menezes et al. 2025) enable fast and water-use-efficient stomatal aperture control (Franks and Farquhar 2007; McAusland et al. 2016; Raissig et al. 2017).

Yet, despite the innovative nature and humanitarian importance of the grass leaf, little is known regarding the processes that generate and coordinate the distinct leaf tissues and cell types to build a functional photosynthetic organ. This is partly due to difficulties inherent to grasses as model systems, like their size, their massive genomes, and their resistance to being transformed by *Agrobacterium tumefaciens* (Raissig and Woods 2022). As a consequence, a lack of tissue-specific reporter lines prevented the description of the transcriptomic signatures of distinct grass leaf tissues and cell types using cell sorting and bulk RNA-sequencing approaches, as was done in the eudicot model system *Arabidopsis thaliana* (eg Brady et al. 2007; Wuest et al. 2010; Adrian et al. 2015). In recent years, the advance of single-cell RNA-sequencing (scRNA-seq) has facilitated the generation of transcriptomes at single-cell resolution and clustering of cells with similar transcriptomic signatures (Grones et al. 2024). These technologies do not require the isolation of specific cell types or tissues using laser microdissection or fluorescence-activated cell sorting (FACS) followed by bulk RNA-seq. Instead, dissociation of tissues and pairing of single plant cells (protoplasts) with beads containing bead-specific barcodes (either in droplets or plates) allows for the generation of single-cell cDNA libraries, massive parallel sequencing of single cell populations, and rapid demultiplexing of reads into single-cell transcriptomes. Strikingly, single-cell transcriptomics in plants repeatedly showed the capability to detect most if not all expected cell types of a given organ and, in addition, revealed cellular heterogeneity and developmental transitions within a tissue of diverse plant species (eg Efroni et al. 2016; Denyer et al. 2019; Nelms and Walbot 2019; Ryu et al. 2019; Zhang et al. 2019, 2021; Satterlee et al. 2020, 2023; Lopez-Anido et al. 2021; Ortiz-Ramírez et al. 2021; Xu et al. 2021, 2025; Sun et al. 2022; Guillotin et al. 2023; Lee et al. 2023, 2025; Swift et al. 2024; Yuan et al. 2024; Demesa-Arevalo et al. 2026).

**Figure 1:**
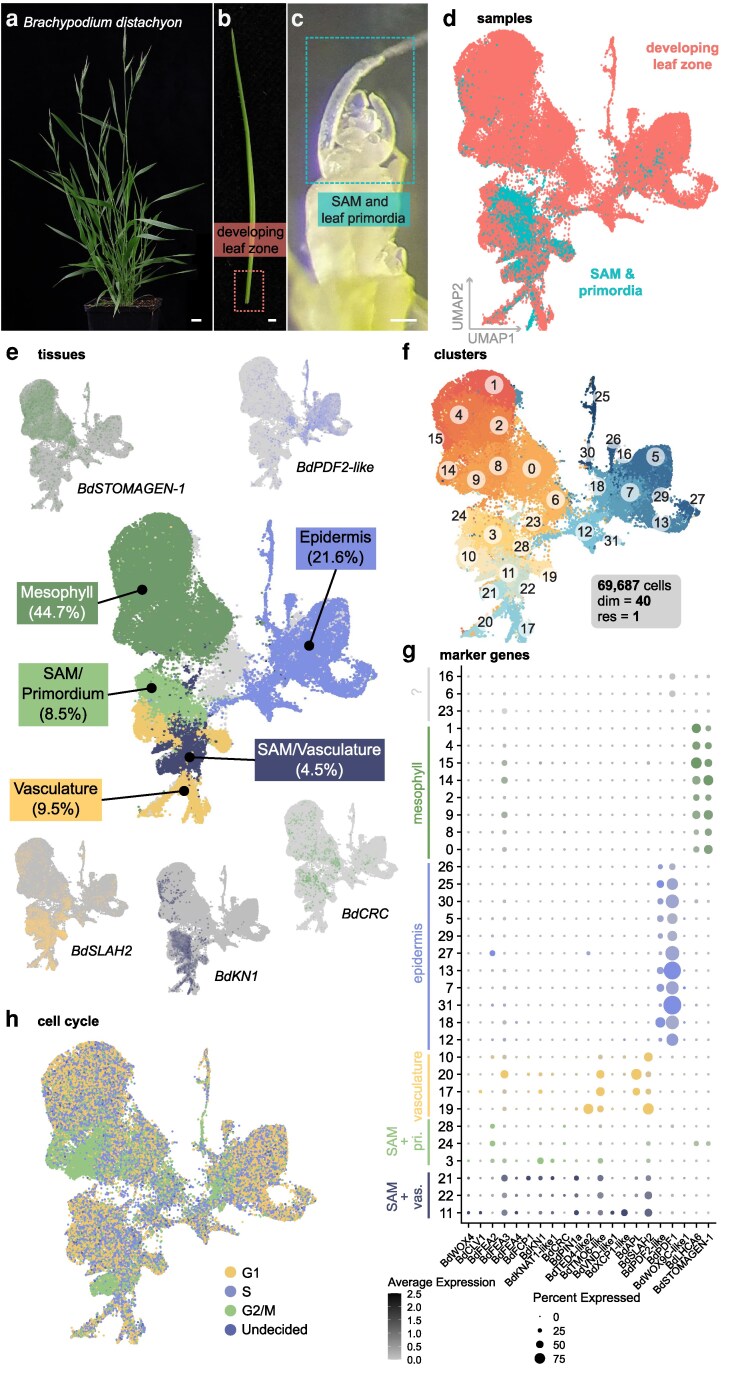
Single-cell atlas of the developing *B. distachyon* leaf from vegetative shoot apex to mature tissues. (a) *Brachypodium distachyon* accession Bd21-3 flowering plant. Image digitally cropped. Scale bar, 1 cm. (b) Young, pulled-out, and not yet unrolled leaf used to isolate the leaf developmental zone (indicated with box). Image digitally cropped. Scale bar, 1 mm. (c) Dissected shoot apex with shoot apical meristem (SAM), leaf primordia, and young outgrowing leaves. Collected tissue is highlighted with a box. Image digitally cropped. Scale bar, 0.1 mm. (d) Whole dataset UMAP plot indicating the tissue-origin of the cells (SAM and primordia in blue, developing leaf zones in red). (e) Whole dataset UMAP plot with tissue annotations (percentage of total cells in brackets) and exemplary marker gene feature plots next to each tissue annotation. N = 69,687 cells. (f) Whole dataset UMAP plot with numbered Seurat clusters. The number of total cells, dimensions, and resolution are indicated. (g) Dot plot showing expression of marker genes in the clusters. Dot size represents the percentage of cells within a cluster that express the gene, and color saturation represents expression strength. Tissues are color-coded as in (e). (h) Whole dataset UMAP plot with color indicating the cell cycle stage of each cell. Also see Figures S1–S3.

Despite the generation of single-cell atlases in (domesticated) grasses, their resistance to transformation remains a significant problem to confirm the clusters identified *in silico* also *in planta* with reporter genes or to generate CRISPR/Cas9-mediated gene knock-outs of cluster-specific genes with a putative regulatory function. We therefore work with the wild Pooideae model grass *Brachypodium distachyon* (Fig. 1a). Not only is *B. distachyon* small in size and easy to grow with limited space, but it is also quite amenable to *A. tumefaciens*-mediated transformation compared to most other grasses, even without access to large-scale transformation facilities. Furthermore, research on wild grass likely reveals higher gene diversity, as wild grasses did not suffer from domestication-induced loss of genetic diversity (Jayakodi et al. 2024).

Grass leaf development is initiated at the vegetative shoot apical meristem (vSAM). In the emerging primordium, the stem cell factor *KNOTTED1* (*KN1*) is downregulated, auxin maxima contribute to primordium outgrowth (O’Connor et al. 2014, 2017; Johnston et al. 2015; Robil and McSteen 2023), and a WUSCHEL-HOMEOBOX module drives medio-lateral leaf expansion (Conklin et al. 2020; Satterlee et al. 2023). In leaf primordia, the ground meristem forms the vasculature and mesophyll, and the protodermal stem cells form all epidermal cell types (Dresselhaus et al. 2025). In the epidermis, protodermal stem cells initially proliferate symmetrically before an asymmetric, transverse patterning division forms a small apical and a larger basal cell (Stebbins and Shah 1960a; Nunes et al. 2020; Berg and Raissig 2025). Mediolateral patterning of the semi-clonal epidermal cell files is determined by antagonistic transcription factor (TF) programs. Grass SPEECHLESS (SPCH) homologs together with INDUCER OF CBF EXPRESSION1 (ICE1) determine stomatal cell file identity (Raissig et al. 2016; Wu et al. 2019; Zhou et al. 2025), whereas SQUAMOSA PROMOTER BINDING PROTEIN-likes (SPLs) establish hair cell (HC) file identity (Kong et al. 2021; Li et al. 2021). In HC files, the apical HC precursors directly form prickle hair cells or macrohairs (Nunes et al. 2023). In stomatal files, the apical guard mother cells (GMCs) elongate and laterally deploy the mobile grass MUTE homolog to establish subsidiary mother cell (SMC) identity in neighboring cells (Raissig et al. 2017; Wang et al. 2019a). SMCs then polarize, and an asymmetric longitudinal division forms the proximal subsidiary cell (SC) (Facette et al. 2015; Zhang et al. 2022). Finally, a symmetric longitudinal division that involves cell-autonomous MUTE function forms the guard cell (GC) pair (Spiegelhalder et al. 2024), before FAMA and SCREAM2 (SCRM2) induce the stomatal pore and guide dumbbell GC morphogenesis (Raissig et al. 2016; Wu et al. 2019; McKown et al. 2023; Wang et al. 2025).

Importantly, even after primordium outgrowth, developing grass leaf blades maintain a spatiotemporally separated developmental leaf zone, where all divisions, cell elongations, and morphogenetic processes occur in a strict base-to-tip developmental gradient (Stebbins and Shah 1960b; Nelson and Dengler 1997; Perico et al. 2022; Berg and Raissig 2025; Dresselhaus et al. 2025). Here, we leveraged this peculiarity of grass leaf development to isolate developing leaf zones of 3-week-old *B. distachyon* plants, which were dissociated, and single-cell transcriptome libraries were built using the droplet-based 10x Genomics platform. In addition, we isolated shoot apices, including vSAMs and early leaf primordia. The overall aim of this study was to build a single-cell atlas of developing grass leaves, which will allow for the dissection of single tissues, lineages, or cell types (eg stomatal cells). We generated a single-cell atlas from shoot apex to mature grass leaf cell types, consisting of almost 70k cells. Marker gene analysis readily identified the 4 major tissues (ie, shoot apical stem cells, vasculature, mesophyll, and epidermis). *In silico* isolation and re-analysis of the epidermal cluster identified all cell lineages of the abaxial epidermis. Multiplexed whole-mount RNA fluorescence in situ hybridization (FISH) confirmed the identified clusters *in planta*. Subsetting of the stomatal lineage and pseudotime analysis of the developing GCs solidly recapitulated previously described developmental modules and identified developmental-stage-specific marker genes. Analysis of homologs of previously reported genetic programs involved in epidermal patterning allowed us to pinpoint clusters that will produce pavement cells, a cell type that is widely unexplored. Single-cell gene regulatory network (GRN) analysis of the stomatal lineages revealed specific targetomes of the co-expressed yet functionally divergent TFs BdMUTE and BdFAMA in GMCs, indicating that our atlas can resolve cell-type- and developmental stage-specific GRNs. As a proof-of-concept, we identified a role for *BdGRAS32* in dividing leaf cells and suggested a role for *BdPME53-like*-mediated processes in stomatal patterning. Together, our high-quality dataset will facilitate the identification of marker genes and GRNs in the developing leaf of *B. distachyon* and can serve as a comparative single-cell gene expression resource for research on both wild and domesticated grass leaves.

## Results

### A single-cell transcriptomic atlas of the developing grass leaf

While some genes involved in the development of the grass leaf have already been investigated (Dresselhaus et al. 2025), much remains unexplored regarding the genetic programs that form and coordinate the diverse cell types of the leaf. Therefore, we generated a single-cell transcriptomic atlas of shoot apices and developing leaves using the wild grass model *B. distachyon* (Fig. 1a) to identify genetic modules that accommodate the development of distinct grass leaf tissues and cell types.

We harvested 2 different tissue samples. First, young, not yet unrolled leaves were extracted from the embracing sheath of the older leaf, and the lowest 3–5 mm were collected for 7 independent “leaf developmental zone” libraries (Fig. 1b, Table S1). Previous microscopy analyses and bulk RNA-seq datasets of the bottom 5 mm of the developing *B. distachyon* leaf suggested that these samples potentially contained all developmental stages and tissues of the leaf (Zhang et al. 2022; Nunes et al. 2023). In addition, we dissected shoot apices, including young leaf primordia, and generated 2 independent “SAM and primordia” libraries (Fig. 1c, Figure S1k and l, Table S1). Then, cell wall digestion released protoplasts (Figure S1a–c), which were used for droplet-based 10x Genomics single-cell RNA-sequencing (scRNA-seq). The resulting datasets, generated at different time points and locations (Table S1), were demultiplexed using Cell Ranger and subsequently filtered to exclude cells with high ambient RNA content, high mitochondrial (>5%) or chloroplast (>10%) RNA content, too few or too many genes and transcripts detected (<1,250 and >50,000 unique molecular identifiers (UMIs); <500 and >10,000 features) and removed doublets to generate a high-quality dataset (see Methods). All 9 datasets were then merged to create the final dataset containing 69,687 cells (expressing 35,584 out of 39,068 genes; Table S1, Table S2; see Methods). Dimensionality reduction and Seurat-based clustering (Satija et al. 2015; Butler et al. 2018; Stuart et al. 2019; Hao et al. 2021, 2024) with a resolution of 0.8 revealed 29 clusters (Figure S1d). Some batch effects were observed (Figure S1k), and we used semi-supervised integration using the STACAS R package (Andreatta and Carmona 2021; Andreatta et al. 2024) to decrease the influence of batch effects. For this, the non-corrected merged dataset was annotated by identifying major tissues using known marker genes from the literature (Figure S1d–j). A subset including SAM and vasculature cells was further analyzed to aim for more detailed annotation of the cells in the subset (Figure S1g–i), and the labels were then transferred to the full dataset (Figure S1g and j). This cell labelling served as the basis for STACAS integration to generate the final dataset, in which the clusters were annotated based on marker expression (Fig. 1e–g). Cells from the “SAM and primordia” libraries were primarily found in the young, developing clusters matching the tissue-origin and the biological context of the libraries (Fig. 1d, Figure S1l).

There was considerable overlap between the vasculature and the shoot apex possibly because the vasculature develops first and originates from the inner ground meristem that constitutes most cells of the leaf primordia (Dresselhaus et al. 2025) and/or because more mature vascular cells did not protoplast well or were filtered out due to cell size limits of our protocol (Fig. 1e and g, Figure S2a–t and z). We were, however, able to detect distinct expression patterns of shoot apex and early vasculature markers (Fig. 1e and g, Figure S2a–t and z). A *FON2-LIKE CLE PROTEIN 1* (*FCP1*) reporter was recently shown to be strongly expressed in the SAM and weaker in the leaf primordial epidermis in barley (Vardanega et al. 2025), while in maize, in situ-hybridization showed it to be more specific to leaf primordia and less in the SAM (Je et al. 2016). We see overlap of *BdFCP1* expression with cells expressing SAM markers (Figure S2d) but also detect expression beyond the early meristem marker *BdKNOTTED1* (*BdKN1*, in maize *ZmKN1* (Smith et al. 1992); Fig. 1e, Figure S2k). This indicates that the expression pattern of *BdFCP1* may be a mix of those detected in barley and maize, spanning both SAM and early leaves. Additionally, spatial transcriptomics and imputed data of stem cell marker homologs in barley further helped in resolving the stem cell clusters (Figure S3a–f and h–k; Demesa-Arevalo et al. 2026). *HvCLV1* is expressed in outer vSAM layers and cells of the leaf primordia (Vardanega et al. 2025), which seems to be similar in *B. distachyon* (Figure S2e, Figure S3a and b). *CRABS CLAW* (*BdCRC*, in barley *HvCRC*) is a marker for leaf primordial tissues (Yamaguchi et al. 2004; Vardanega et al. 2025) and helped to indicate leaf primordial clusters in *B. distachyon* (Figure S2i, Figure S3c and d). *PIN-FORMED 1a* (*BdPIN1a*, *HvPIN1a*) and *PIN1b* (*BdPIN1b*, *HvPIN1/HvPIN1b*) are expressed in leaf primordia and inner meristem, respectively (Figure S3h–k; Kirschner et al. 2018; Fusi et al. 2024). However, in our dataset, we detected more widespread *BdPIN1b* expression and could not rely on this marker for cluster annotations (Figure S3h).

To annotate the epidermal tissue clusters, we used the well-established *PROTODERMAL FACTOR 1* (*BdPDF1*) and *BdPDF2* and relied on the expression pattern of a *B. distachyon* transcriptional reporter of *BdHDG2-like*, which is one of the earliest leaf epidermal markers in *A. thaliana* (Peterson et al. 2013) (Fig. 1e and g; Figure S2u and v; Figure S3l and m). To annotate the mesophyll tissue clusters, we used photosynthetic genes (eg *BdLHCA6*; Figure S2x) and the mesophyll-expressed epidermal patterning factor *BdSTOMAGEN-1* (Figure S2y).

Finally, we used homologs of *A. thaliana* cell cycle marker genes to assign cell cycle phases to the dataset (Fig. 1h; https://github.com/raissig-lindner-lab/Berg-et-al_2025_ScSeq). Four domains of mitotically active cells (ie, G2-to-M phase cells) were identified; 1 domain lies in the shoot apex and vasculature clusters and expresses *BdWOX4* and *BdPIN1a*, 1 lies in the mesophyll, and 2 in the epidermis (Fig. 1g and h, Figure S2a and j).

Taken together, we generated a 70k-cell containing single-cell transcriptomic atlas of developing grass leaves from the shoot apex to the mature leaf. A specific identity could be assigned to most clusters, dividing cells could be clearly identified, and meristematic tissues could be distinguished from developing vasculatures.

### Subsetting the epidermis identifies distinct epidermal lineages

**Figure 2:**
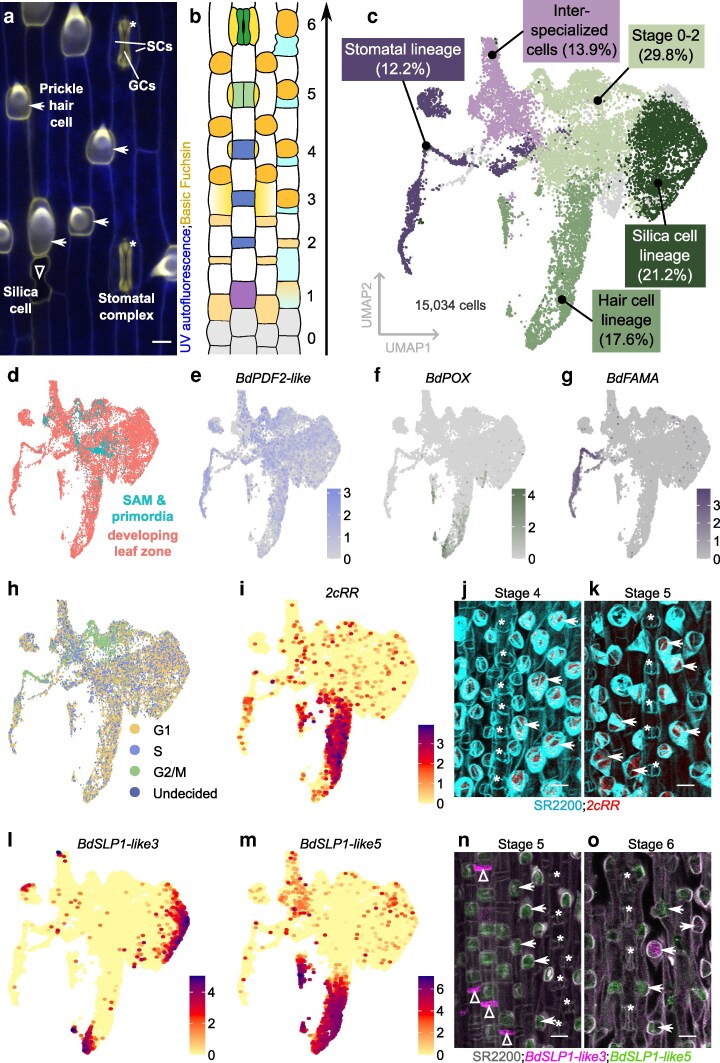
Subsetting the epidermis identifies epidermal lineages and marker genes. (a) Confocal image of the mature abaxial leaf epidermis of *B. distachyon*. Stomatal complexes are marked with asterisks, prickle hair cells with arrows, and silica cells with triangles. Blue is cell wall autofluorescence, and yellow is lignin stained by basic fuchsin (image adapted from Berg and Raissig 2025). (b) Schematic of leaf epidermal developmental stages in *B. distachyon*. Protodermal stem cells (stage 0) acquire cell file identities (stage 1) and divide asymmetrically to form an apical guard mother cell (GMC) or hair cell (HC) lineage precursor and an interstomatal or inter-hair cell (stage 2). Inter-hair cells above leaf veins can divide again asymmetrically to form silica cell precursors. A GMC-derived signal initiates subsidiary mother cell (SMC) identity in lateral neighboring cells (stage 3). SMCs divide asymmetrically to form subsidiary cells (SCs) (stage 4). GMCs divide symmetrically to form 2 guard cells (GCs) (stage 5), which then differentiate into dumbbell-shaped GCs (stage 6). (c) Epidermis UMAP plot with epidermal cell type annotations (percentage of total cells in brackets). N = 15,034 cells. (d) Epidermis UMAP plot indicating the tissue-origin of the cells (SAM and primordia in blue, developing leaf zones in red). (e) Epidermis UMAP feature plot of epidermal marker *BdPDF2-like*. (f) Epidermis UMAP feature plot of HC marker *BdPOX*. (g) Epidermis UMAP feature plot of stomatal marker *BdFAMA*. (h) Epidermis UMAP plot with color indicating the cell cycle stage of each cell. (i) Epidermis UMAP feature plot of a two-component response regulator (*2cRR*). (j and k) Whole-mount RNA-FISH images of the *2cRR* (red) at developmental stages 4 (j) and 5 (k). Cell walls stained with SR2200 (cyan). Asterisks indicate stomatal complexes and arrows indicate HCs. (l) Epidermis UMAP feature plot of *BdSILIPLANT1-like3* (*BdSLP1-like3*). (m) Epidermis UMAP feature plot of *BdSLP1-like5*. (n and o) Whole-mount multiplex RNA-FISH images of *BdSLP1-like3* (magenta) and *BdSLP1-like5* (green) at developmental stages 5 (n) and 6 (o). Cell walls stained with SR2200 (gray). Asterisks indicate stomatal complexes, arrows indicate HCs, and triangles indicate silica cell precursors. Color legends in the UMAP feature plots show expression strength. Scale bars,10 µm. Also see Figure S4.

We then subsetted the epidermal lineages and specifically reclustered and reanalyzed the developing grass leaf epidermis from protodermal stem cells to maturing and differentiating epidermal cell types (Fig. 2c, Figure S4a and b). The mature abaxial leaf epidermis in *B. distachyon* consists of linear cell files that form either stomatal complexes or prickle hair cells (HCs), which are spaced by at least 1 pavement cell (Fig. 2a). Above leaf veins, additional transverse divisions form silica cells below HCs. The grass leaf epidermis develops in a strictly linear manner, with 7 well-defined stages of stomatal development used as reference points throughout this manuscript (Fig. 2b; Stebbins and Shah 1960b; Raissig et al. 2016, 2017; Nunes et al. 2020; Berg and Raissig 2025).

We used tissue-of-origin (ie the developmentally younger “SAM & primordia” libraries; Fig. 2d) and epidermal marker genes to assign stage 0–2 protodermal cells and stomatal and HC lineage identities (Fig. 2c–g, Figure S4d–n) and used cell cycle markers to assign cell cycle phases (Fig. 2h). Notably, there were 2 clusters with many mitotically active cells (Fig. 2h). The first one is likely the cells that divide during stages 0 and 2 of epidermal development without any clear indication of future cell fates. The second one is specific to the stomatal lineage and likely represents dividing GMCs (see below).

We then compared clusters of interest to all other epidermal clusters to identify differentially expressed marker genes (DEGs) for different cell types (Table S4). Putative marker genes were chosen according to specific expression patterns in a given cell type or stage and were filtered to exclude protoplast-affected genes (ie, bulk RNA-sequencing data of non-protoplasted versus protoplasted tissues; Figure S4c, Table S5). Subsequently, we used whole-mount RNA fluorescence in situ hybridization (HCR^TM^ RNA-FISH, Dirks and Pierce 2004; Choi et al. 2018) to verify the expression pattern of candidate marker genes *in planta*. A two-component response regulator (*2cRR*) was confirmed to be a marker for HCs at stages 4 and 5 of epidermal development (Fig. 2i–k).

For silica cells, the only known marker gene was the sorghum *SbSILIPLANT1* (*SbSLP1*), a gene suggested to precipitate silica in leaf silica cells (Kumar et al. 2020; Ayieko et al. 2023). Homologs of *SbSLP1* showed specific expression patterns (Figure S4o–w); *BdSLP1-like1, 2, 3,* and *4* showed strong *in silico* expression in silica cells and mature HC stages, while *BdSLP1-like5, 6, 7,* and *9* seemed specific to HCs, with *BdSLP1-like5* appearing during earlier stages already (Fig. 2l and m; Figure S3o–w). We, therefore, chose *BdSLP1-like3* as a marker for silica cells and mature HCs and *BdSLP1-like5* as a marker for HCs only. Multiplexed RNA-FISH showed that at stage 5, *BdSLP1-like3* was expressed in silica cell precursors, whereas *BdSLP1-like5* was exclusive to HCs (Fig. 2n). During stage 6, *BdSLP1-like3* stopped being expressed in silica cells and instead started to be expressed in HCs, while *BdSLP1-like5* expression decreased (Fig. 2o).

In conclusion, subsetting the epidermal lineages successfully identified the HC and silica cell lineages, which were confirmed *in planta* using multiplexed whole-mount RNA-FISH of identified marker genes.

### Isolation of the stomatal lineage clusters identifies both grass stomatal cell types

**Figure 3:**
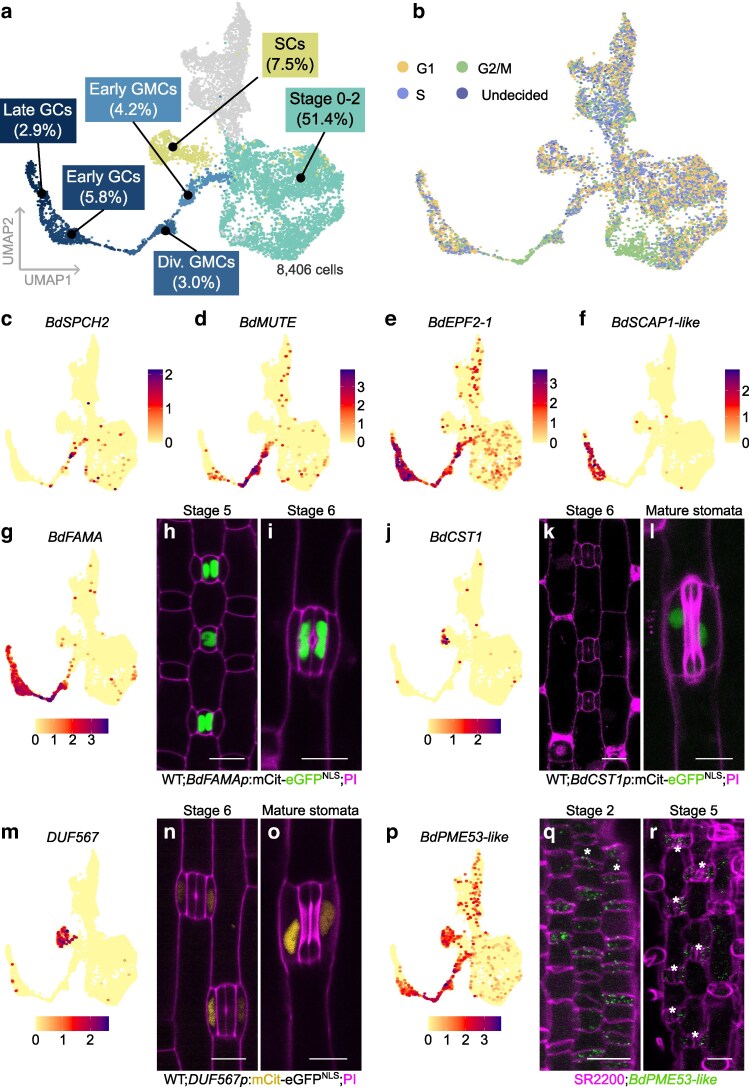
Spatiotemporal development of the stomatal lineage. (a) Stomatal lineage UMAP plot with stomatal cell types and stage annotations (percentage of total cells in brackets). N = 8,406 cells. (b) Stomatal lineage UMAP plot with color indicating the cell cycle stage of each cell. (c) Stomatal lineage UMAP feature plot of early stomatal lineage marker *BdSPCH2*. (d) Stomatal lineage UMAP feature plot of guard mother cell (GMC) marker *BdMUTE*. (e) Stomatal lineage UMAP feature plot of guard cell (GC) lineage marker *BdEPF2-1*. (f) Stomatal lineage UMAP feature plot of maturing GC marker *BdSCAP1-like*. (g) Stomatal lineage UMAP feature plot of GC lineage marker *BdFAMA*. (h–i) Expression of *BdFAMAp:mCitrine-eGFP^NLS^* in developmental stages 5 (h) and 6 (i). (j) Stomatal lineage UMAP feature plot of mature subsidiary cells (SCs) marker *BdCST1*. (k and l) Expression of *BdCST1p:mCitrine-eGFP^NLS^* in developmental stage 6 (k) and mature SCs (l). (m) Stomatal lineage UMAP feature plot of SC marker *DUF567*. (n and o) Expression of *DUF567p:mCitrine-eGFP^NLS^* in developmental stage 6 (n) and mature SCs (o). Cell walls stained with propidium iodide (PI, magenta) in all reporter images. (p) Stomatal lineage UMAP feature plot of *BdPECTIN METHYLESTERASE 53-like* (*BdPME53-like*). (q and r) Whole-mount RNA-FISH images of *BdPME53-like* (green) at developmental stages 2 (q) and 5 (r). Cell walls stained with SR2200 (magenta). Asterisks indicate stomatal cell files (q) or stomatal complexes (r). Color legends in the UMAP feature plots show expression strength. Scale bars, 10 µm. Also see Figure S5.

Graminoid stomatal morphology is highly derived, with 2 dumbbell-shaped GCs that are flanked by lateral SCs (Fig. 2a and b). To decode the transcriptional dynamics of the developing grass stomatal lineages, we used known marker genes to isolate all stomatal lineage cells from the epidermal cluster (*n* = 8,406 cells, Figs. 2g and 3a, Figure S4g–n, Figure S5a and b). For example, the sequentially acting bHLH TFs *SPEECHLESS* (*SPCH*), *MUTE FAMA* and *STOMATAL CARPENTER1 (SCAP1)*, and the guard-cell-lineage associated *EPIDERMAL PATTERNING FACTOR 2–1* (*BdEPF2-1*) were used to assign the GC lineage (Fig. 3c–g, Figure S5d–h; Raissig et al. 2016, 2017; McKown et al. 2023). The generation of a *BdFAMA* transcriptional reporter clearly demarcated the developmental stages expressing *BdFAMA* (Fig. 3h and i). Furthermore, cell cycle phase assignment once again revealed actively dividing cells at stage 0–2 and the symmetrically dividing GMCs (Fig. 3b), which also express *BdMUTE* and *BdFAMA* (Fig. 3d and g).

The SCs were harder to identify as only a single, very late expressed SC-specific marker is known to date, *ZmCST1* (Wang et al. 2019b). In our first datasets, we did not detect the expression of *BdCST1* and could therefore not rely on this gene to map SCs. To *bona fide* identify the SC population, 2 of the 7 developmental zone libraries were produced from the *sid*/*bdmute-1* genotype that lacks SCs (Figure S1l, Table S1; Raissig et al. 2017). Indeed, comparison of the 2 wild-type (WT) libraries and the 2 SC-lacking *sid/bdmute-1* libraries processed and sequenced at the same time readily identified a cluster strongly enriched for WT cells, suggesting this cluster represents the SCs (Figure S5c). The most recent libraries contained many more SCs and, consequently, enabled the detection of *BdCST1* transcripts *in silico* in this exact cluster (Fig. 3j). A *BdCST1* transcriptional reporter suggested that only very mature SCs express this marker (Fig. 3k and l).

Differential gene expression analysis then identified stomatal marker genes. A *DUF567* gene showed strong and almost exclusive *in silico* expression in the SC cluster (Fig. 3m). Within the epidermis, a transcriptional reporter for *DUF567* was indeed expressed very specifically in SCs from early stage 6 to mature stomatal complexes (Fig. 3n and o), earlier than *BdCST1*. Furthermore, the pectin methylesterase *BdPME53-like* was suggested to be expressed in the developing GC and SC lineages (Fig. 3p). Indeed, a signal was detected from stage 2 GMCs to stage 5 GCs and in SCs from recruitment until maturing stomata (Fig. 3q and r).

In conclusion, we successfully identified and confirmed the GC and SC lineages, found SC and GC markers, and cloned promoters specific to the SC lineage.

### Pseudotime trajectory of the grass guard cell lineage identifies stage-specific marker genes

**Figure 4:**
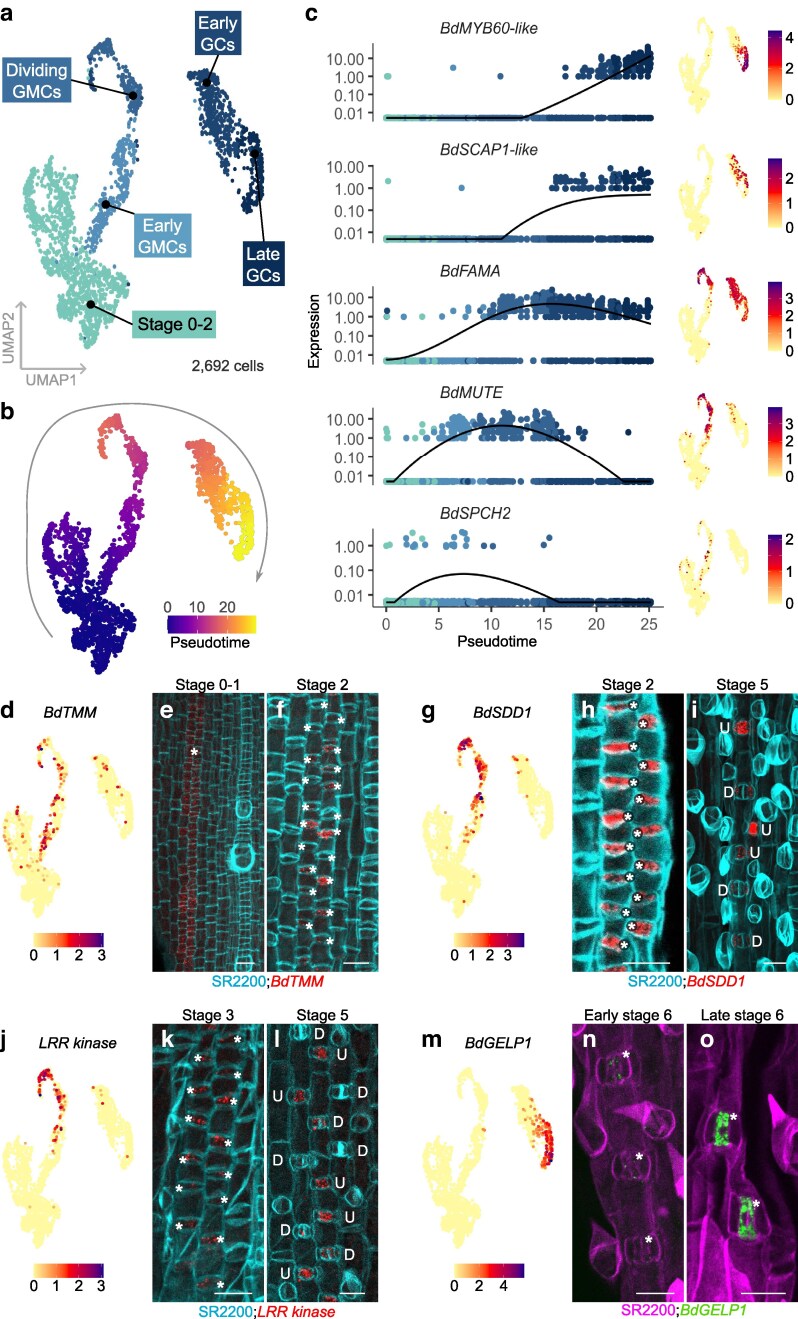
Sequential gene expression in the stomatal guard cell lineage. (a) UMAP plot with developing guard cell (GC) lineages; developmental stages are color-coded and annotated. N = 2,692 cells. (b) GC lineage UMAP plot with color indicating pseudotime. Arrow indicates developmental trajectory; heatmap indicates pseudotime stages. (c) Dot plot showing expression of marker genes along pseudotime. GC lineage UMAP feature plots of the respective genes are shown on the right. (d) GC lineage UMAP feature plot of *BdTOO MANY MOUTHS* (*BdTMM*). (e and f) Whole-mount RNA-FISH images of *BdTMM* (red) at developmental stages 0-1 (e) and 2 (f). Cell walls stained with SR2200 (cyan). Asterisks indicate stomatal cell files (e) and stomatal complexes (f). (g) GC lineage UMAP feature plot of *BdSTOMATAL DENSITY AND DISTRIBUTION 1* (*BdSDD1*). (h and i) Whole-mount RNA-FISH images of *BdSDD1* (red) at developmental stages 2 (h) and 5 (i). Cell walls stained with SR2200 (cyan). Asterisks indicate guard mother cells (GMCs). “U” indicates undivided and “D” indicates divided GMCs. (j) GC lineage UMAP feature plot of *LEUCINE RICH REPEAT KINASE* (*LRR kinase*). (k and l) Whole-mount RNA-FISH images of *LRR kinase* (red) at developmental stages 3 (k) and 5 (l). Cell walls stained with SR2200 (cyan). Asterisks indicate guard mother cells (GMCs). “U” indicates undivided and “D” indicates divided GMCs. (m) GC lineage UMAP feature plot of *BdGDSL ESTERASE/LIPASE1* (*BdGELP1*). (n and o) Whole-mount RNA-FISH images of *BdGELP1* (green) at early (n) and late (o) developmental stage 6. Cell walls stained with SR2200 (magenta). Asterisks indicate stomatal complexes. Color legends in the UMAP feature plots show the expression strength of the gene of interest. Scale bars, 10 µm. Also see Figure S6.

Even though spatial (and spatiotemporal) information is lost in droplet-based scRNA-seq approaches, the temporal dynamics can be inferred using pseudotime analysis, particularly for lineages with sufficient a priori knowledge on developmental transitions like the GC lineage. Thus, we subsetted the GC lineage based on the stomatal bHLH TFs (*n* = 2,692 cells, Fig. 3c, d, and g, Fig. 4a, Figure S6a and b). Pseudotime analysis revealed the developmental gradient from early divisions at stage 0–2 towards fully differentiated GCs (Fig. 4a and b). Plotting the expression of known markers of different stages across the pseudotimeline confirmed the accuracy of the developmental GC trajectory (Fig. 4c).

We then selected several sequential marker genes along this trajectory to confirm the developmental timeline from protodermal stem cells to maturing GCs *in planta*. The stomatal *BdTOO MANY MOUTHS* (*BdTMM*) co-receptor was suggested to be expressed from the earliest stages in the GC lineage (Fig. 4d, Figure S6c). RNA-FISH revealed that *BdTMM* was truly expressed in very early, stage 0–1 stomatal files preceding the transverse asymmetric patterning division (Fig. 4e). After this division, the signal was mostly restricted to GMCs and disappeared after stage 2 (Fig. 4f). A homolog of the stomatal peptidase STOMATAL DENSITY AND DISTRIBUTION1 (SDD1; Von Groll et al. 2002) showed strong *in silico* expression in young and dividing GMCs (Fig. 4g). Indeed, we found strong RNA-FISH signal for *BdSDD1* in stage 2 GMCs up until the symmetric division, after which it quickly disappeared, strongly matching the *in silico* expression prediction of our dataset (Fig. 4h and i). A *LEUCINE-RICH-REPEAT PROTEIN KINASE* (*LRR kinase*) showed a similar, yet slightly later *in silico* expression pattern compared to *BdSDD1* (Fig. 4j). Much like *BdSDD1*, the signal of the *LRR kinase* probe was detected from stage 2 GMCs and abruptly disappeared in stage 5 as soon as the GMC had completed its division (Fig. 4k and l). Lastly, the *GDSL ESTERASE/LIPASE1* gene (*BdGELP1*) was suggested to be a mature GC marker gene (Fig. 4m), which was confirmed by RNA-FISH probes against *BdGELP1 in planta* (Fig. 4n and o).

We also performed pseudotime analysis for the HC lineage, to test if it can produce a useful trajectory with less a priori knowledge (Figure S6d–f). Indeed, we found the expected sequential expression of the few currently known HC developmental marker genes (Figure S6g), suggesting that our dataset, paired with pseudotime analysis, can reveal marker genes and developmental regulators of any epidermal lineage.

### Grass pavement cells are highly heterogenous

In contrast to stomatal cells, HCs, or silica cells, no marker genes have been reported for pavement cells in grasses. We, therefore, used stomatal lineage-related genes to identify interstomatal cell clusters, which are stomatal lineage-derived pavement cells, and attempted to use these as a proxy to identify general pavement cell markers.

In *A. thaliana*, the ERECTA-YODA-MITOGEN-ACTIVATED PROTEIN (MAP) kinase (MAPK) signaling cascade ensures spacing of stomata by at least 1 pavement cell through the down-regulation of stomatal bHLH TFs upon reception of EPF1 and EPF2 peptide ligands that are secreted by developing stomatal complexes (reviewed in Herrmann and Torii 2021; Chua and Lau 2024). In grasses, the inhibitory role of the pathway in epidermal cell file patterning seems to be conserved. For example, EPF ligands were shown to affect stomatal density (eg Caine et al. 2019; Jangra et al. 2021; Karavolias et al. 2023) and *yoda1* mutants in *B. distachyon,* and barley caused within-file clustering of specialized cells (Abrash et al. 2018; Liu et al. 2022).

**Figure 5:**
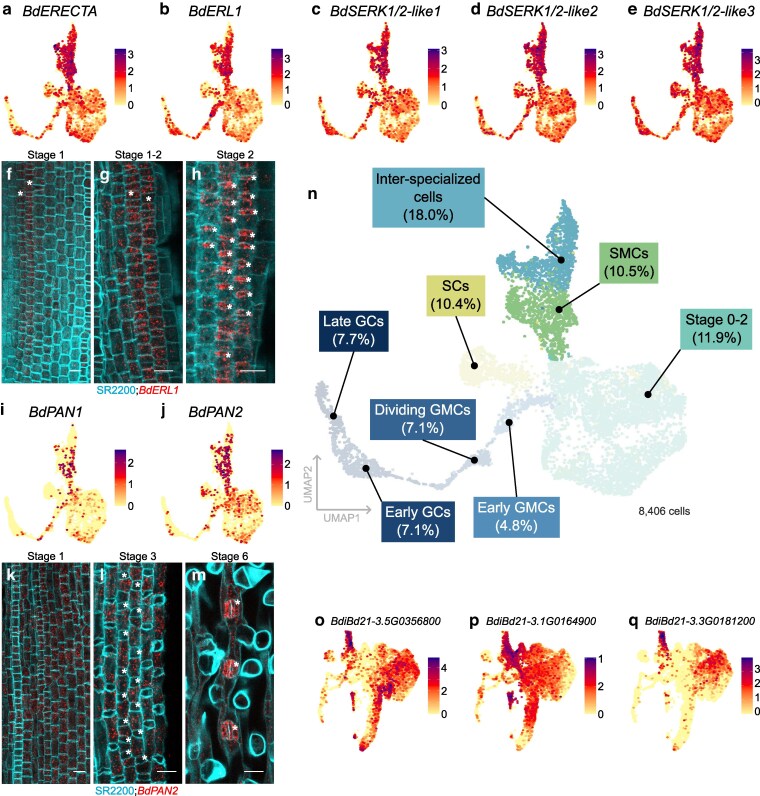
Analysis of interstomatal pavement cells as a proxy to identify transcriptomic pavement cell features. (a–e) Stomatal lineage UMAP feature plot of *BdERECTA* (a), *BdERECTA-LIKE1* (*BdERL1*) (b), *BdSOMATIC EMBRYOGENESIS RECEPTOR KINASE1/2-like1* (*BdSERK1/2-like1*) (c), *BdSERK1/2-like2* (d), and *BdSERK1/2-like3* (e). (f–h) Whole-mount RNA-FISH images of *BdERL1* (red) at developmental stage 1 (f), stage 1 to 2 (g), and stage 2 (h). Cell walls stained with SR2200 (cyan). Asterisks indicate stomatal cell files (f and g) and guard mother cells (GMCs, H). (i–j) Stomatal lineage UMAP feature plot of *BdPANGLOSS1* (*BdPAN1*) (i) and *BdPAN2* (j). (k–m) Whole-mount RNA-FISH images of *BdPAN2* (red) at developmental stage 1 (k), 3 (l), and 6 (m). Cell walls stained with SR2200 (cyan). Asterisks indicate GMCs (l) and stomatal complexes (m). (n) Stomatal lineage UMAP plot with stomatal cell type and stage annotations (percentage of total cells in brackets). Same plot as in Fig. 3a but with interstomatal cells and subsidiary mother cells (SMCs) labeled and highlighted; *n* = 8,406 cells. (o–q) Epidermis UMAP feature plots of marker genes of the interstomatal cell clusters. Color legends in the UMAP feature plots indicate expression strength. Scale bars, 10 µm. Also see Figure S5.

We hypothesized that the ERECTA-YODA-MAPK signaling pathway is preferably active in interstomatal pavement cells and analyzed the *in silico* expression patterns of *B. distachyon* homologs of the inhibitory signaling pathway. As expected, *BdEPF2-1* and *BdEPF2-2* ligands are primarily expressed in the GC lineage (Fig. 3e, Figure S5h). *BdYDA1/2* and the putative downstream MAPKs *BdMKK4/5-like1*, *BdMKK4/5-like2*, *BdMPK3-like,* and *BdMPK6-like* were expressed rather ubiquitously, yet a slight enrichment in the topmost clusters was observed (clusters 3, 6, 8, and 10; Figure S5b and i–n). Similarly, *BdERECTA*, *BdERECTA-LIKE1* (ERL1) and the 3 co-receptors *SOMATIC EMBRYOGENESIS RECEPTOR KINASE-likes* (*BdSERK-likes*) were strongly and preferentially expressed in clusters 3, 6, 8, and 10 (Fig. 5a–e, Figure S5b), suggesting that these not yet annotated cells could potentially be the interstomatal or inter-specialized cells (highlighted cells, Fig. 5n). Indeed, RNA-FISH against *BdERL1* confirmed its expression in stomatal cell files from very early stages of stomatal development through to GMCs and interstomatal cells after the transverse patterning division at stage 2 (Fig. 5f–h).

Such inter-specialized cells can also be stomatal SMCs. However, no exclusive SMC markers are known. The main SMC identity factor *BdMUTE* is not transcriptionally expressed in SMCs but rather translocates from GMCs to SMCs (Raissig et al. 2017; Spiegelhalder et al. 2024), and many of the genes affecting SMC polarization and division are not specific to SMCs (Figure S5o–t; Berg and Raissig 2025). Nonetheless, the 2 polarization factors *BdPANGLOSS1* (*BdPAN1*) and *BdPAN2* (Cartwright et al. 2009; Zhang et al. 2012, 2022) appeared to be quite strongly expressed in the putative interstomatal cells (Fig. 5i and j). *BdPAN1* showing the strongest expression in clusters 8 and 6, as well as the presence of *BdPOLAR* there, suggests that these clusters corresponded to SMCs (Fig. 5i and n, Figure S5b and o). RNA-FISH confirmed *BdPAN2* expression in interstomatal cells, SMCs, and pavement cells in hair cell files (Fig. 5k and l). Beyond stage 2 and 3, *BdPAN2* seemed to be strongly expressed in SCs and, later, in GCs, a pattern that matched its described function in SC shape and *in silico* expression in our atlas (Sutimantanapi et al. 2014) (Fig. 5m). Together, this suggested that clusters 3, 6, 8, and 10 are inter-specialized cells, with clusters 6 and 8 potentially being enriched for SMCs (Fig. 5n, Figure S5b, Fig. 2c).

We then attempted to identify marker genes in the inter-specialized cells that might be general pavement cell markers. When mapping some of these putative pavement cell markers onto the epidermal dataset, we got either very broad, non-specific expression patterns (Figs. 5o, and 2c) or markers for what appears to be developmentally younger cells (Figs. 5p, and 2c) or marker genes that are expressed alongside the hair or silica cell domain in addition to interstomatal cells (Figs. 5q, and 2c).

Together, this indicated that pavement cells are highly heterogenous and their transcriptional identity might vary significantly depending on whether they stem from the stomatal, the hair cell, or the silica cell lineage. This might explain why pavement cells do not form specific, readily identifiable lineage clusters in grasses.

### Gene regulatory network analysis identifies key transcription factors as the main regulons in the stomatal lineage

**Figure 6:**
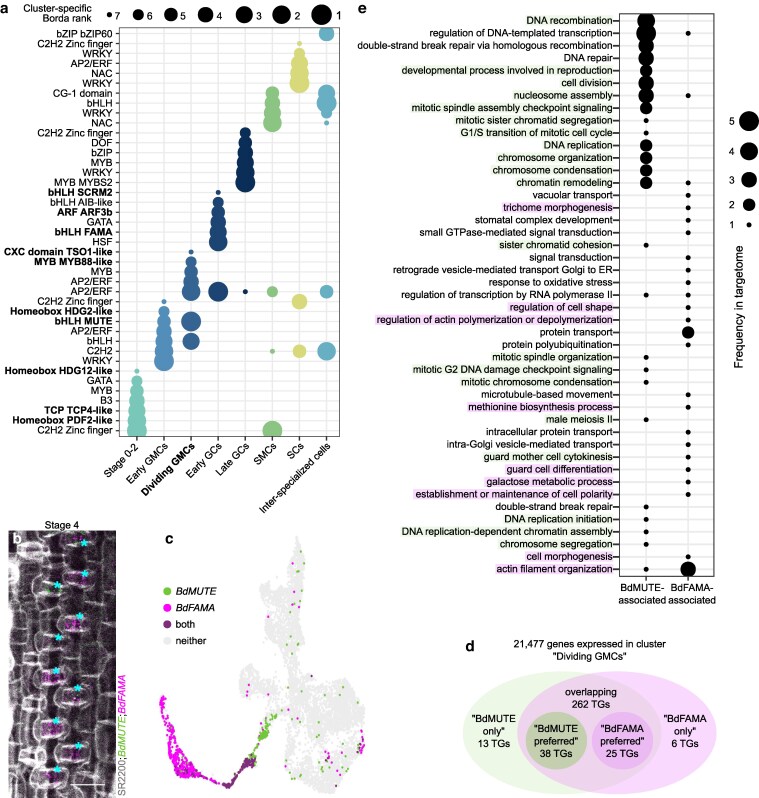
Gene regulatory network analysis of the stomatal lineages. (a) Dot plot showing the top 7 regulons (= transcription factors (TFs)) per cluster. TFs highlighted in bold have already been described in the context of leaf development. (b) Whole-mount multiplex RNA-FISH image of *BdMUTE* (green) and *BdFAMA* (magenta) at developmental stage 4. Cell walls stained with SR2200 (gray). Asterisks indicate stomatal complexes. Scale bar, 10 µm. (c) Stomatal lineage UMAP feature plot of *BdMUTE* (green) and *BdFAMA* (magenta). Cells expressing both genes are colored dark purple, cells expressing neither are in gray. (d) Venn diagram of target genes (TGs) of BdMUTE (green) and BdFAMA (magenta) in the “Dividing GMCs” cluster. 13 TGs are exclusive to BdMUTE (“BdMUTE only”), 6 TGs are exclusive to BdFAMA (“BdFAMA only”), and 262 genes are targeted by both TFs. Of these, 38 show preferential co-expression with BdMUTE (logarithmic weight ratio >3) and 25 show preferential co-expression with BdFAMA (logarithmic weight ratio <3). (e) Dot plot showing the frequency of the top 25 GO terms for “BdMUTE-associated” TGs (“BdMUTE only” and “BdMUTE preferred”) and “BdFAMA-associated” TGs (“BdFAMA only” and “BdFAMA preferred”). GO terms associated with cell division are marked in green, and GO terms associated with cell morphogenesis are marked in magenta. Also see Figure S7.

To identify the main TF hubs and their putative target genes in the stomatal clusters, we conducted gene regulatory network (GRN) analysis of the stomatal subset using MINI-EX (version 3; Ferrari et al. 2022; Staut et al. 2026). The GRN analysis revealed different TF regulons that may be key to regulating the developmental progression of cell types at different developmental stages. Among the top 7 most relevant TFs for each stomatal lineage cluster, we found several that were already implicated in leaf and stomatal development (Fig. 6a, Figure S7a, Table S6).

Within the early cluster of stage 0–2, we found *BdPROTODERMAL PATTERNING FACTOR 2-like* (*BdPDF2-like*), which is closely related to *AtPDF2* and defines L1/epidermal fate in the *A. thaliana* shoot apex (Abe et al. 2003) in second position (Fig. 6a, Figure S7b, Table S6). A homolog of the leaf morphogenesis TF *AtTEOSINTE BRANCHED 1, CYCLOIDEA, PROLIFERATING CELL FACTOR1/2 4* (*AtTCP4*; Palatnik et al. 2003), *BdTCP4-like*, was found in the third position (Fig. 6a; Figure S7c, Table S6).

*BdMUTE* is the top regulon in the dividing GMC cluster in line with its role in regulating GMC cell division orientation (Fig. 6a; Spiegelhalder et al. 2024). *BdTSO1* and *BdMYB88-like*, whose *A. thaliana* orthologs were found to be involved in the GMC to GC transition (Lai et al. 2005; Simmons et al. 2019), are in sixth and seventh position at this stage (Fig. 6a, Figure S7d and e, Table S6). *BdFAMA*, shown to regulate GC differentiation and pore formation (McKown et al. 2023), was the third most relevant regulon in early GCs (Fig. 6a, Table S6), and its putative heterodimerization partner *BdSCRM2* was in seventh position in early GCs (Fig. 6a, Table S6; Raissig et al. 2016; McKown et al. 2023).

In conclusion, single-cell GRN analysis revealed core TF hubs in accordance with their implied function from molecular and genetic analysis during grass stomatal development and provides a valuable resource to find key regulators in the developmental stages of interest.

### Distinct targetomes of BdMUTE and BdFAMA in guard mother cells

The MINI-EX output also yields target genes (TGs) per regulon (targetomes), if known TF motifs and a list of all genes that contain such motifs in their cis-regulatory regions (generated with the FIMO tool (Grant et al. 2011)), are used as input. This enabled cluster-specific analyses of putative downstream genetic programs for each identified regulon per cluster.

The 2 bHLHs *BdMUTE* and *BdFAMA* overlap in their expression windows in GMCs both *in silico* and *in planta* (Fig. 6b and c), yet have distinct functions in regulating division orientation and GC differentiation, respectively (McKown et al. 2023; Spiegelhalder et al. 2024). Therefore, they provide an excellent example to evaluate putative differences in the targetomes of 2 closely related TFs with the same binding motif (CANNTG bHLH motif; Voronova and Baltimore 1990; Atchley and Fitch 1997) yet distinct functional roles. In the cluster “Dividing GMCs” (clusters 14 and 16, Fig. 6a, Figure S5b), 21,477 genes are expressed, of which 262 appeared as TGs for both TFs, 13 were exclusive to *BdMUTE,* and 6 were exclusive to *BdFAMA* (Fig. 6d, Table S7). Each TF-TG combination was assigned a weight value describing the strength of co-expression between the 2 genes. To identify preferential TGs, we calculated the ratio of weight_BdMUTE_ by weight_BdFAMA_ and filtered for TGs that had a logarithmic weight ratio of higher than 3 to obtain genes that have a stronger association with *BdMUTE* and lower than −3 for genes that are more linked to *BdFAMA*. This resulted in an additional 38 preferential TGs for *BdMUTE* and 25 preferential TGs for *BdFAMA* (Fig. 6d). We then explored the functional annotations of exclusive and preferential TGs using gene ontology (GO) terms (Fig. 6d and e). Notably, the *BdMUTE* TGs are more likely to be involved in mitotic processes (Fig. 6e, green), whereas the *BdFAMA* targetome seemed to be rather associated with cell wall biology and cellular morphogenesis (Fig. 6e, magenta), consistent with their genetically determined functions. The functionally divergent TG-set enrichment of *BdMUTE* and *BdFAMA* suggested that our atlas allows for the discovery of cell-type and TF-specific gene networks in a highly targeted manner.

### From division control to stomatal density—functional analysis of two developmental marker genes

**Figure 7:**
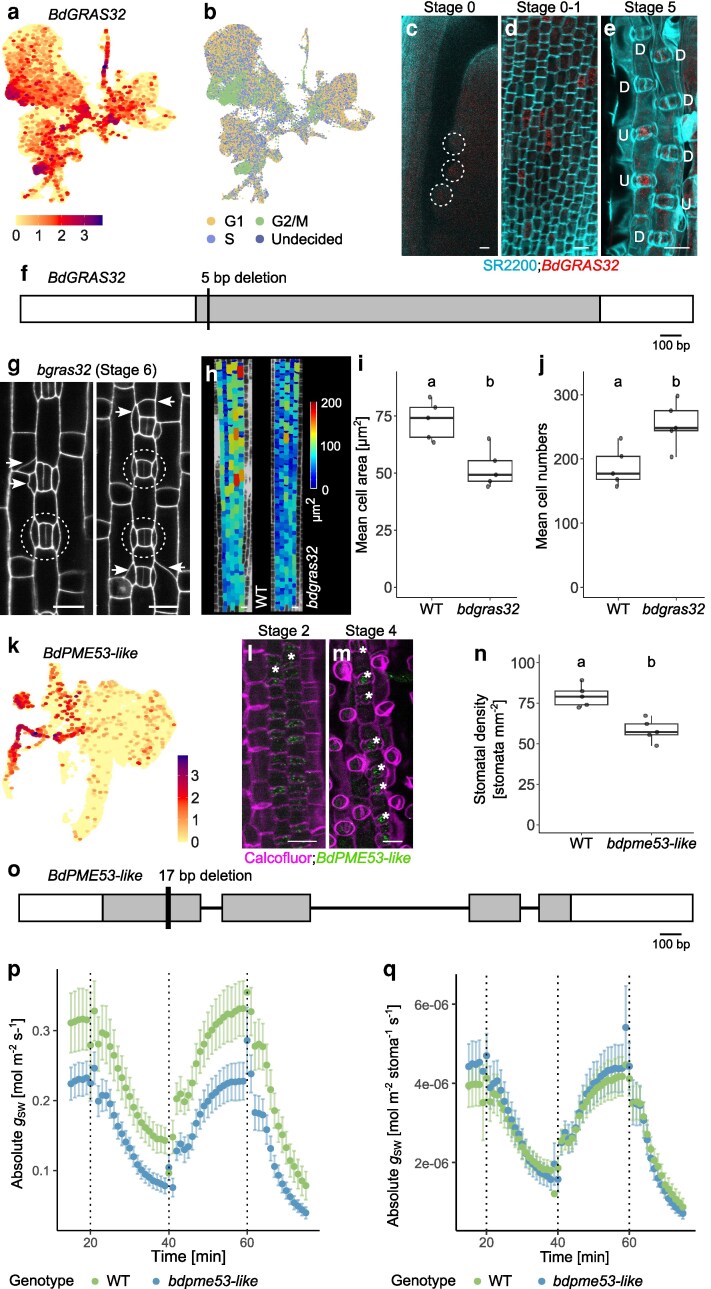
Functional analysis of selected marker genes. (a) Whole dataset UMAP feature plot of *BdGRAS32*. (b) Whole dataset UMAP plot with color indicating the cell cycle stage of each cell. Same plot as in Fig. 1h. (c–e) Whole-mount RNA-FISH images of *BdGRAS32* (red) in leaf primordia (c, circled), epidermis developmental stage 0-1 (d), and 5 (e). Cell walls stained with SR2200 (cyan). “U” indicates undivided and “D” indicates divided guard mother cells (GMCs). Scale bars, 10 µm. (f) Gene model of *BdGRAS32* with the mutation of the *bdgras32* mutant is indicated. (g) Mutant phenotype of *bdgras32* at developmental stage 6. Arrows indicate misdivisions, ellipses indicate wild type (WT)-like stomatal complexes. Scale bars, 10 µm. (h) Representative MorphoGraphX heat map images of WT and *bdgras32*. Color indicates cell area. Scale bar, 50 µm. (i) Mean cell area of WT and *bdgras32*; *n* = 5 individuals per genotype. (j) Mean cell numbers of WT and *bdgras32*; *n* = 5 individuals per genotype. (k) Epidermis UMAP feature plot of *BdPECTIN METHYLESTERASE 53-like* (*BdPME53-like*). (l and m) Whole-mount RNA-FISH images of *BdPME53-like* (green) in developmental stage 2 (m) and 4 (n). Cell walls stained with Calcofluor (magenta). Asterisks indicate stomatal cell files (m) and stomatal complexes (n). Scale bars, 10 µm. (n) Stomatal density of WT and *bdpme53-like*; *n* = 5 individuals per genotype. (o) Gene model of *BdPME53-like* with the mutation of the *bdpme53-like* mutant indicated. (p and q) Absolute stomatal conductance (*g*_SW_) of WT (green) and *bdpme53-like* (blue) per leaf area (p) and per stoma (q). Standard error is indicated by error bars. Color legends in the UMAP feature plots indicate expression strength. Significant differences calculated with an unpaired, two-sided Student's *t*-test (*P* < 0.5) are indicated by differing letters.

We selected 2 marker genes for mutant analysis to show how this dataset can help to identify players in leaf or stomatal development. First, a GRAS TF, *BdGRAS32*, which is the orthologue of the rice TF *DWARF AND LOW-TILLERING (DLT*)/*OsGRAS-32* (Xie et al. 2019), was identified as a putative cell division factor throughout leaf development as one of the transcription factors upregulated in mitotically active cells, with more than 67% of *BdGRAS32*-positive cells (expression > 1) being categorized as G2/M phase cells (Fig. 7a and b, Table S4). RNA-FISH verified that the gene was already expressed in very young leaf primordia at the shoot apex and in the earliest stages of epidermal development (Fig. 7c and d). Finally, strong expression could also be detected in GMCs shortly before the division at stage 5 (Fig. 7e). This suggested a role in regulating cell division during *B. distachyon* leaf development. To investigate this, we generated a *bdgras32* CRISPR mutant (Fig. 7f). We detected misdivisions with seemingly misplaced, additional division planes around GCs and in SCs (Fig. 7g). Imaging and segmentation of the developing leaf epidermis (stage 1–2) of wild type and *bdgras32* mutants (Fig. 7h) showed significantly higher cell numbers and, as a consequence, smaller cells in *bdgras32* compared to wild type (Fig. 7i and j). Additionally, *bdgras32* above-ground tissue showed significantly lower dry weight than WT, although its fresh weight was not significantly different (Figure S7g and h). Together, we propose that *BdGRAS32* represses cell division capacity in the developing *B. distachyon* leaf.

To functionally describe a stomatal marker gene, we chose to gene edit *BdPME53-like* (Fig. 7k and o), which we established as a marker for the developing GC and SC lineage based on its upregulation in stomatal lineage clusters and in situ RNA-FISH expression analysis (Fig. 3p–r, Fig. 7l and m; Table S4). Additionally, this gene was a putative target of the BdMUTE and BdFAMA TFs based on our GRN analysis (Table S7). We hypothesized that mutating *BdPME53-like* could affect cell wall properties and interfere with biomechanical aspects of stomatal opening and closing due to its functional annotation as a pectin methylesterase. Indeed, leaf-level gas exchange measurements indicated a decrease in stomatal opening, represented by lower stomatal conductance to water vapor (*g*_SW_, Fig. 7p). Analysis of stomatal anatomy, however, showed that *bdpme53-like* formed significantly fewer stomata (Fig. 7n). By correcting the gas exchange values for averaged stomatal density per genotype, *g*_SW_ was no longer lower in the mutant on a per-stoma basis (Fig. 7q), suggesting that the observed physiological phenotype might not be due to impaired stomatal function but a decrease in stomatal density in *bdpme53-like*.

In conclusion, our proof-of-concept functional validation of 2 genes strongly suggested that the *B. distachyon* single-cell leaf atlas can be leveraged to identify developmental factors that shape the functional anatomy of the most important photosynthetic powerhouse for ecosystems and humanity alike.

### An online gene expression viewer for the community

To make this dataset readily accessible, we created an expression browser tool to visualize the expression of any *B. distachyon* gene of interest in our leaf atlas (Figure S7f). We generated different tabs so that gene expression can be visualized with feature plots of the whole dataset, the epidermal subset, or the stomatal subset (https://shiny.ips.unibe.ch/). Next to the feature plot, the expression viewer displays a UMAP plot with annotated tissues for reference. In addition, we computed a ggPlantmap (Jo and Kajala 2024) visualization of the *bona fide* assigned tissues and cell types for an eFP-browser-like experience. A download function generates an overview of all tissues, the epidermal and the stomatal subset (feature plot of the gene and UMAP plot with tissue identities), and the ggPlantmap overview (Figure S7f). We hope that this “*B. distachyon* leaf gene expression viewer” will be used by the research groups working primarily on *B. distachyon* and the extended grass developmental research community.

## Discussion

The genetic programs that pattern and coordinate the development of different tissues and cell types in grass leaves are still largely unexplored. In the past, gene discovery mainly relied on mutant screens or analysis of homologs of genes involved in leaf development in *A. thaliana*. The advent of single-cell/nucleus transcriptomics nowadays facilitates gene discovery, gene network, and gene co-expression analysis in an unbiased, genome-wide, and model system-independent manner (Grones et al. 2024).

Here, we provide a single-cell transcriptomic leaf atlas of the wild model grass *B. distachyon* that comprises almost 70k cells from the vegetative shoot apex to the leaf developmental zone (Fig. 1). We used known markers of different tissues, cell types, and stages to spatially and pseudotemporally resolve cluster identities (Figs. 1–5). While this study focused mostly on the leaf epidermis, we were also able to identify distinct expression patterns of developmental markers within the shoot apex and vasculature (Figures S2 and S3). We predict that in-depth analysis of the vascular and mesophyllar subsets will allow the discovery of developmental regulators along the spatiotemporal trajectories of these tissues, even though some of the very large cells or tissues with massive secondary cell wall deposition and modification might not have been quantitatively captured.

Furthermore, we were not able to identify one rather enigmatic grass leaf epidermal cell type, the bulliform cells. Little is known about their developmental timing and genetic programs. We hypothesize that we might not have captured these cells in our libraries due to their large cell size and/or physiological properties. Bulliform cells are rather long and bulging cells of the adaxial epidermis, and there is a high probability that they were size filtered as the 10x Genomics platform has a cell size limit of around 40 µm (Grones et al. 2024). In addition, bulliform cells are functionally associated with leaf rolling and water release under arid conditions (Kellogg 2015; Matschi et al. 2020). Therefore, these cells might experience rather high cell turgor under well-watered conditions and are thus likely to burst when cell walls are digested to release protoplasts. A different sequencing platform designed to accommodate bigger cells (eg BD Rhapsody) or the use of nuclei rather than protoplasts can overcome cell size restrictions and protoplast bursting in the future. Similarly, mature pavement cells might also be missing due to size exclusion, bursting, or suboptimal protoplasting. The strong presence of *ER*, *ERL1,* and *SERKs* in certain clusters of the epidermis, however, indicated that we were able to obtain at least younger, and therefore smaller, inter-specialized pavement cells (Fig. 5). However, we were not able to find pavement cell-specific markers. We hypothesize that pavement cell transcriptomes in the strictly linearly patterned grass leaf epidermis tend to resemble their respective sister cells within a cell file, due to their shared origin. Therefore, we expect the young pavement cells to cluster close to their specialized neighbor cells, as appears to be the case for the inter-specialized cells we analyzed (Fig. 5).

Next, pseudotime analysis nicely recapitulated the developmental transitions of the GC lineage and HC lineage. For both lineages, however, stage-specific marker genes were known before, in particular for the GC lineage, where many of the early core developmental regulators are identified and genetically characterized (Raissig et al. 2016; McKown et al. 2023; Spiegelhalder et al. 2024). This suggested pseudotime analysis is a very powerful tool. Yet, such an analysis will also forcibly yield a trajectory that might be biologically nonsensical. Some a priori knowledge of the biological context is required, and biologically meaningful, ontogenetically related cell lineages should be isolated prior to pseudotime analysis.

We then implemented single-cell-resolved GRN analysis (using MINI-EX), which enabled the investigation of the targetomes of selected TFs within or across selected clusters. The comparison of 2 closely related TFs, *BdMUTE* and *BdFAMA*, which are both expressed in the dividing GMCs but regulate GMC division and GC differentiation, respectively (McKown et al. 2023; Spiegelhalder et al. 2024), showed that we are able to distinguish these 2 biological processes within a selected cluster based on the targetomes of the 2 distinct regulons (Fig. 6). Importantly, this method will allow comparative approaches between different scRNA-seq datasets of closely and distantly related species that use the same TFs for distinct stomatal morphologies and compositions (ie grasses and eudicots) as the targetome is more likely to be modified by evolution than the regulon (ie, TF) itself (Andrews et al. 2023; Marand et al. 2023; Amundson et al. 2025). Despite its elegance and power, there are limitations to the GRN analysis applied here. Firstly, it relies on transcriptional expression of a TF in the cell type of interest. This suffices for the regulatory analysis within that cell type, but mobile, non-cell autonomously acting TFs are excluded. *BdMUTE*, for example, is transcriptionally expressed and has a functional role in the GMCs, but its primary role is moving from GMCs to neighboring cells and establishing the SC lineage (Raissig et al. 2017; Spiegelhalder et al. 2024). Our GRN approach can therefore only decipher putative targets of BdMUTE within the GMCs, but not in the SMCs. In addition, the targetome analysis is based on genes being co-expressed with a regulon and will therefore only yield target genes that are positively regulated by the regulon, but might miss downregulated target genes.

To demonstrate how our dataset can be used to find additional components of leaf development, we selected 2 genes with a specific expression pattern for functional analysis. Mutations in *BdGRAS32*, which was specifically expressed in mitotically active cells, showed smaller, more numerous cells and division defects (Fig. 7g–j). This confirms a similar phenotype found in rice mutants, where *dlt/osgras-32* mutants showed increased leaf width and higher cell numbers by regulating cyclin-related genes (Xie et al. 2019). It is therefore likely that *BdGRAS32* also restricts cell division through the regulation of cell cycle genes in *B. distachyon*. Mutations in the stomata-specific gene *BdPME53-like* affected stomatal density and, consequently, leaf-level gas exchange (Fig. 7k–q). This suggests a role of *BdPME53-like*-mediated cell wall modifications during stomatal development, which may influence stomatal lineage patterning in the epidermis. In *A. thaliana*, the mutant of the closest related gene, *AtPME53*, had slightly increased stomatal length, width, and stomatal density, yet decreased stomatal aperture (Wu et al. 2022). As we observed an opposing phenotype regarding stomatal density, more work will be required to determine how *AtPME53* and *BdPME53-like* influence stomatal lineage development and why this may lead to opposite phenotypes.

In conclusion, we present a spatio-pseudotemporally resolved single-cell atlas of a developing grass leaf from leaf primordia at the shoot apex to mature leaf tissues in the wild grass model *B. distachyon*. This dataset can be used to identify and select genes in a targeted manner for downstream functional analyses to further decode leaf developmental processes in the most important plant family for humanity–the grasses.

## Materials and methods

### Materials availability

Recombinant DNA, transgenic reporter lines, and mutant lines are available upon request. All materials generated and used in this study are listed in Table S9.

### Plant material and growth conditions

The *B. distachyon* Bd21-3 ecotype was used as the wild type (WT), and the *sid* (also known as *bdmute-1*) line described in (Raissig et al. 2017) was used as the mutant line when creating the single-cell transcriptomic atlas. Seeds were dehusked and then vernalized and stratified for 2 d in water (dark, 4 °C). Then, they were planted on soil (4 parts Einheitserde CL ED73 (Einheitserdewerke, Werkverband e.V., Sinntal-Altengronau, Germany) and 1 part Vermiculite) and grown in a greenhouse or growth chamber with an 18 h light:6 h dark cycle (photosynthetically active photon flux density (PPFD) 200–400 μmol m^−2^ s^−1^; day temperature 28 °C, night temperature 22 °C) as described in (Nunes et al. 2022).

For the imaging of *B. distachyon* transcriptional reporter lines *DUF567p:mCitrine-eGFP^NLS^* and *BdFAMAp:mCitrine-eGFP^NLS^*, as well as the *bdpme53-like* mutant plants for phenotyping, were grown on soil as described above. *BdHDG2-likep:^3XNLS^eGFP*, *BdCST1p:mCitrine-eGFP^NLS,^* and the *bdgras32* mutant plants (plus their wild type control) were grown on MS plates (Murashige & Skoog (Duchefa Biochemie B.V, Haarlem, The Netherlands), 1% Agar (w/v), pH 5.7; Haas and Raissig 2020) and imaged 1 wk (reporter) or 6 d (mutant) after germination.

*Hordeum vulgare* (barley) cultivar Golden Promise plants for the smRNA-FISH experiment were grown as described in (Demesa-Arevalo et al. 2026). To reiterate: Plants were grown in soil (Einheitserde CL ED73) in 96-well trays with 4 g/L Osmocote Exact Hi.End 3–4 M, 4th generation (ICL Group, Ladenburg, Germany) under long day conditions (16 h light at 20 °C and 8 h dark at 16 °C). Grains were pre-germinated at 4 °C for 4 d on a layer of wet paper in Petri dishes, and vegetative shoot apical meristems were collected 11 d after sowing in soil.

For the barley reporter line, *HvFCP1p:VENUS-H2B* (Vardanega et al. 2025), which is in the barley cultivar Golden Promise Fast (Gol et al. 2021) background, plants were grown under the same conditions as described above, with the pre-germination treatment lasting 3 d.

### Method details

#### Generation of protoplasts and single-cell RNA-Sequencing

Single cells (protoplasts) were extracted from 2 tissue types of 3 wk old, soil-grown plants. 7 samples were generated from the leaf developmental zone for 5 WT and 2 *sid* samples collected at different dates and locations using plants grown in either greenhouse or growth chamber conditions (details on the samples in Tables S1 and S2). As the laboratory moved from Heidelberg to Bern during the course of this study, 3 of the 5 WT leaf samples were generated in Bern, because we wanted to test if the new growth conditions and location generate significantly different single-cell transcriptomes for the developing *B. distachyon* leaves. For the leaf developmental zone datasets, 15 young, not yet unrolled leaves were carefully pulled from the enveloping sheath (Fig. 1b), each leaf from a different plant individual, and the lowest 3–5 mm were cut and collected in a petri dish with 3 ml Milli-Q water on ice. 2 WT samples were used to generate the vegetative shoot apex with leaf primordia of 8 and 10 individuals per sample, respectively. The plant was cut off right above the soil level, and the older leaves were carefully removed under a binocular microscope to reach the shoot apical area. A cut was made right below the youngest outgrowing leaf, as indicated with a dashed line in Fig. 1c, and the tissue piece was collected in a petri dish with 3 ml Milli-Q water on ice. No further pre-treatment was done with the samples. Protoplasts were generated using a modified version of the protocol described in (Ortiz-Ramírez et al. 2018), with detailed steps as follows: The samples were vacuum infiltrated in water in a vacuum desiccator for 5 min and then incubated for 45 min to hydrate the cell walls (RT, dark, 90 rpm shaking). In the meantime, enzymes (Cellulase “Onozuka RS” (Duchefa, final concentration 2.4% (w/v)) and Macerozyme R-10 (Duchefa, final concentration 0.4% (w/v))) were added to 3 ml of the washing solution (D-Mannitol (Roth, final concentration 0.4 M), MES hydrate (Sigma-Aldrich, St. Louis, Missouri, United States, final concentration 20 mM), KCl (Sigma-Aldrich, final concentration 20 mM), CaCl_2_ (Sigma-Aldrich, final concentration 10 mM), BSA (Sigma-Aldrich, final concentration 0.1% (w/v), pH adjusted to 5.7 with 1 M Tris-HCl (pH8)). After incubation, the water was carefully removed and replaced by 3 ml enzyme solution and then vacuum-infiltrated for 5 min (this extra vacuum step was not mentioned in the original (Ortiz-Ramírez et al. 2018) protocol). Then the sample was cut into very small pieces using nail scissors (less than 1 mm) and incubated for 90 min (RT, dark, 90 rpm shaking). About 3 mm of the tips of 1 ml pipette tips were cut off, and the protoplast suspension was gently pipetted up and down for 3 min to mechanically release protoplasts. Then the protoplast-containing solution was transferred (cut pipette tip!) and slowly filtered through a 20 µm mesh filter (CellTrics, Sysmex cat. no. 04-004-2325) into a 1.5 ml LoBind tube (Eppendorf, Basel, Switzerland) (on ice). The sample was centrifuged for 3 min (300 x *g*, 4 °C). Two washing steps followed, where the supernatant was removed with an uncut pipette tip without disturbing the pellet, before careful resuspension in 1 ml washing solution and centrifuged again for 3 min (300 x *g*, 4 °C). After 2 rounds of washing, the pellet was resuspended in 500 ml washing solution for the developmental zone samples and 100 ml for the vegetative shoot apex samples. Protoplast viability was assessed by staining with fluorescein diacetate (FDA (Honeywell International Inc., Fluka^TM^, 0.01% (w/v) solution in acetone) for 30 min in the dark (only done for cells that were not used for sequencing). Green fluorescence of viable cells was visualized using an epifluorescence microscope (Leica DM5000B or DM2000 (Leica Microsystems, Wetzlar, Germany)). Cells were counted using a Fuchs-Rosenthal chamber (Brand GmbH + Co KG, Wertheim, Germany) on a DM2000 or DM5000B light microscope (Leica Microsystems) and brought to the sequencing facility for quality control, library preparation, and sequencing (Deep Sequencing Core Facility, Heidelberg University, Germany, and Next Generation Sequencing Platform, University of Bern, Switzerland, respectively). The Chromium Next GEM Single Cell 3′ Reagent Kit v3.1 (10x Genomics, Pleasanton, United States) was used for library preparation, and the libraries were sequenced using different Illumina Short Read sequencing platforms (see Tables S1 and S2).

#### Data preparation and primary analysis of the single-cell RNA-Sequencing data

The Illumina sequencing files were first pre-processed using Cell Ranger (10x Genomics Cell Ranger v7.0.1, Zheng et al. 2017) with default settings. In short, the reference genome *B. distachyon* Bd21-3 v1.2 (obtained from Phytozome: https://phytozome-next.jgi.doe.gov/info/BdistachyonBd21_3_v1_2) was prepared with Cell Ranger *mkref,* and afterwards the libraries were de-multiplexed, barcodes were processed, and gene counts were computed with Cell Ranger *count*. For the 2022 datasets, both libraries (WT and *sid*) were sequenced twice using different sequencing platforms (see Table S1), and the libraries were merged using Cell Ranger *count*. Then, the data was filtered to exclude cells with high ambient RNA content using the SoupX package with default settings (version 1.6.2, Young and Behjati 2020) in RStudio, and the SoupX objects were used to initialize Seurat objects for downstream processing with the Seurat package (version 5.1.0, Satija et al. 2015; Butler et al. 2018; Stuart et al. 2019; Hao et al. 2021, 2024). The following steps were repeated for each of the 9 datasets: To filter low quality cells, those with more than 5% mitochondrial and/or more than 10% chloroplast counts were excluded (mitochondrial and chloroplast genes used in this context are based on blast results from *A. thaliana* genes ((Ward 2001); *B. distachyon* gene accessions list on GitHub: https://github.com/raissig-lindner-lab/Berg-et-al_2025_ScSeq), as well as cells with less than 1,250 or more than 50,000 UMIs (nCount_RNA) and less than 500 or more than 10,000 features (nFeature_RNA). Within-cell expression was normalized with NormalizeData (default settings). FindVariableFeatures (default settings) was used to identify the top 2,000 variable genes in the dataset. Gene expression was normalized across cells with ScaleData (default settings). A principal component analysis (PCA, RunPCA for 50 principal components) followed by Uniform Manifold Approximation and Projection (UMAP, RunUMAP with 30 dimensions) was conducted to reduce dimensionality of the datasets. Potential doublet cells were filtered out using the DoubletFinder package (version 2.0.4, McGinnis et al. 2019) according to the GitHub instructions (https://github.com/chris-mcginnis-ucsf/DoubletFinder). Expected doublet formation rates were estimated using the Chromium Next GEM Single Cell 3ʹ Reagent Kit v3.1 User Guide as reference (10x Genomics, https://assets.ctfassets.net/an68im79xiti/1eX2FPdpeCgnCJtw4fj9Hx/7cb84edaa9eca04b607f9193162994de/CG000204_ChromiumNextGEMSingleCell3_v3.1_Rev_D.pdf). All 9 datasets were subsequently merged without integration. The resulting dataset was again processed using FindVariableFeatures, ScaleData, and RunPCA (100 principal components). FindNeighbors (40 dimensions), FindClusters (resolution 0.8), and RunUMAP (40 dimensions) then yielded 29 clusters. G2-to-M (G2/M) and S phase genes were identified by blasting *A. thaliana* cell cycle genes (Vandepoele et al. 2002); *B. distachyon* gene accessions list on GitHub: https://github.com/raissig-lindner-lab/Berg-et-al_2025_ScSeq), and cell cycle states were assigned using the CellCycleScoring function. Tissue identities were assigned to clusters by comparing expression patterns of known marker genes (Figures S1–S3, Table S3). Epidermis and mesophyll identity was assigned to clusters with epidermal (eg, Figure S1f) and mesophyll (eg Figure S1e) markers (Figure S1j). A subset of the cells containing the clusters 2, 3, 16, 19, 20, 21, 22, and 26 was extracted, and the previously described analysis pipeline was repeated (30 principal components, 25 dimensions, resolution 0.6; Figure S1i). Clusters of the subset were assigned to tissues based on marker gene expression, and the cell labels were transferred to the whole dataset (Figure S1g–j). Semi-supervised integration of the datasets was conducted using STACAS (version 2.4.0; Andreatta and Carmona 2021; Andreatta et al. 2024); Run.STACAS function with 40 dimensions (2,000 anchor features) with the previously assigned tissue identities (Figure S1j) to decrease batch effects across the cell atlas. Clusters were grouped using 40 dimensions and resolution 1. Once again, the same marker genes as before were used to assign tissue and cell type identities on this integrated dataset (Fig. 1, Figures S2 and S3). To obtain and analyze subsets, clusters of interest were extracted and re-clustered. For the epidermal dataset, all clusters labeled as “Epidermis” were subsetted from the whole dataset (30 principal components, 25 dimensions, resolution 1). For the stomatal lineage subset, clusters annotated as “Inter-specialized cells”, “Stage 0-2,” and “Stomatal lineage” were extracted from the epidermal subset (30 principal components, 25 dimensions, resolution 0.8). Cell types/stages were identified and annotated using known marker genes, cell cycle phases, and sample origin (ie, “SAM and leaf primordia” or “leaf developmental zone” datasets) (see Figs. 2–5). Visualization of the single-cell sequencing data was done using Seurat and ggplot2 (version 3.5.2, Wickham 2016). The default assay to use for plotting gene expression was always “RNA” and was changed to “integrated” when analyzing and clustering subsets. The complete R script is available on GitHub: https://github.com/raissig-lindner-lab/Berg-et-al_2025_ScSeq.

#### Pseudotime analysis

For the pseudotime analyses, the GC and HC subsets were extracted by subsetting the “Stage 0-2” clusters and either GC-associated clusters (“GMCs” and “GCs”) or “Hair cell lineage” clusters from the stomatal lineage subset or epidermal subset, respectively (for both subsets: 30 principal components, 25 dimensions, resolution 0.5). The resulting Seurat objects were converted to monocle 3 cell_data_set objects using the SeuratWrappers package (version 0.4.0, Butler et al. 2024). Monocle 3 (version 1.3.7, Qiu et al. 2017a, 2017b; Cao et al. 2019) was used to process the datasets (dimension = 50, resolution = 0.001), to identify and visualize pseudotime trajectories, and compute and visualize the expression of selected marker genes along the pseudotime trajectories. The R script is available on GitHub: https://github.com/raissig-lindner-lab/Berg-et-al_2025_ScSeq.

#### Gene regulatory network and GO term analysis

Gene regulatory network analysis of the stomatal lineage subset was conducted using MINI-EX (version 3.1, Ferrari et al. 2022; Staut et al. 2026). First, input files needed to be created for *B. distachyon* Bd21-3 genome v1.2. The expression matrix, cluster markers, and annotations were extracted from the Seurat object of the stomatal lineage subset clusters 3, 5, 6, 7, 8, 9, 10, 11, 12, 13, 14, and 16 (Figure S5b), annotated with their respective cell type and stage (Fig. 5n), as described on the MINI-EX GitHub page (https://github.com/VIB-PSB/MINI-EX/blob/main/docs/data_preparation.md). The list of *B. distachyon* transcription factors (TFs) was downloaded from the Plant Transcription Factor Database: https://planttfdb.gao-lab.org/index.php?sp=Bdi. The .meme file and .txt information file for *B. distachyon* TF motifs were also downloaded from the Plant Transcription Factor Database: https://planttfdb.gao-lab.org/download.php#bind_motif. A conserved bHLH TF motif (CANNTG, Voronova and Baltimore 1990; Atchley and Fitch 1997) for the stomatal bHLH TFs BdSPCH1, BdSPCH2, BdMUTE, BdFAMA, BdICE1, and BdSCRM2 was manually added to the list. To map the TF motifs to the genome, the *B. distachyon* Bd21-3 genome v1.2 .fasta file was downloaded from Phytozome (https://phytozome-next.jgi.doe.gov/info/BdistachyonBd21_3_v1_2). The FIMO online tool (https://meme-suite.org/meme/tools/fimo, Grant et al. 2011) was used to look for TF binding sites (TFBS) across the genome, which were then manually filtered to obtain a list of target genes (TGs). FIMO TFBS hits across each chromosome were linked to genes by using the chromosomal position information, which is available in the FIMO results and can be linked to the gene position given in the *B. distachyon* Bd21-3 v1.2 .gff file. Only 1 hit per TG and TF was kept, and the thus cleaned TG list was extracted and used as input for MINI-EX.

After all required input files were obtained, MINI-EX was run with default settings (topMarkers was changed to 1,000 instead of 700) and including the motif filtering analysis (the motif filter argument was set to “TF-F-motifs” to use the motifs known for a TF family). Further analysis and visualization of the MINI-EX output files were done in RStudio. The top 7 TFs per cluster were filtered based on their cluster-specific Borda ranking in the MINI-EX rankedRegulons output file. To compare the targetomes of BdMUTE and BdFAMA, the MINI-EX edgeTable output file was filtered for TGs in dividing GMCs and then for those that could be bound by BdMUTE or BdFAMA. The TF-exclusive genes were selected by filtering for genes only bound by either of the TFs. By calculating the ratio of weight_BdMUTE_ (as an indicator of the strength of co-expression between the TF and the TG) by weight_BdFAMA,_ and considering TGs with a weight ratio > 3 as preferentially regulated by BdMUTE and a weight ratio < −3 indicated those genes that may rather be regulated by BdFAMA. The list of TGs that were classified as preferentially regulated by BdMUTE was merged with the BdMUTE-exclusive TGs, and the same was done for BdFAMA. Gene Ontology (GO) terms for biological processes obtained using the R packages Go.db (version 3.20.0, Carlson 2024) and AnnotationDbi (version 1.68.0, Pagès et al. 2024) were linked to the TGs, and for the top 25 GO terms per dataset, their frequency was visualized with ggplot2. Any additional information and details on file preparation and modifications, as well as analysis and visualization of the MINI-EX output files, can be found in the GRN R Script on Github (https://github.com/raissig-lindner-lab/Berg-et-al_2025_ScSeq).

#### Selection of candidate genes

Differential gene expression analysis was conducted in R to find marker genes of different cell types and/or developmental stages using the Seurat FindMarkers function (default settings, Table S4). To find markers in mitotically active cells of the whole dataset, the FindMarkers function was used in the whole dataset, with the clustering feature being the G2/M phase of the cell cycle annotations (Fig. 1h, Table S4). Markers in subclusters of the epidermal subset and stomatal lineage subset were detected based on Seurat clusters (Figures S4b and S5b). Additionally, for the G2/M phase markers, the file was filtered to only include transcription factors that are upregulated in this cell cycle phase. Thus, obtained candidates were screened manually by checking their gene expression feature plots. Of this selection, only genes were considered for downstream analysis that were not upregulated (fold change > 3) in the protoplasted bulk RNAseq data compared to non-protoplasted (see below). A subset of those genes representing a diverse panel of cell types and developmental stages was chosen for HCR RNA-FISH and transcriptional reporters to confirm their expression and thus cluster annotation *in planta* (selected candidate genes from this approach are marked green in Table S4). The selected genes were linked to known orthologues and functionally annotated using information obtained from Phytozome gene annotations (Goodstein et al. 2012), InterPro (Blum et al. 2025), PaperBLAST (Price and Arkin 2017), and the OMA browser (Altenhoff et al. 2021).

#### Bulk RNA-Sequencing datasets to compare protoplasted and non-protoplasted tissue

To assess gene expression changes induced by protoplasting, total RNA was extracted from both protoplasted and non-protoplasted developmental leaf zones of 3 wk old Bd21-3 (WT) plants. Protoplasts were prepared from 10 developmental zones according to the protocol above, and 5 leaf zones were used for the non-treated (ie non-protoplasted) control (3 biological replicates per condition). RNA extraction was performed with the RNeasy micro kit (QIAGEN GmbH, Hilden, Germany) with on-column DNase digest following the manufacturer's instructions. RNA concentration was assessed with the Qubit RNA High Sensitivity (HS) Assay Kit (Invitrogen, Thermo Fisher Scientific, Massachusetts, United States). RNA quality check, library preparation, and sequencing on a NextSeq 550 platform (Illumina Inc., San Diego, United States) were conducted at the Deep Sequencing Core Facility of Heidelberg University (Germany).

Pre-processing of the bulk RNA-seq results was done on https://usegalaxy.org/. After quality control of the sequencing results with FastQC (Andrews 2010), the bulk transcriptomes were mapped to the Bd21-3 v1.1 genome (https://phytozome-next.jgi.doe.gov/info/BdistachyonBd21_3_v1_1) with bowtie2 (Langmead et al. 2009, 2019; Langmead and Salzberg 2012). Further processing was done using R (version 4.5.1, R Core Team and R Foundation for Statistical Computing 2025) in RStudio (version 2024.12.01, RStudio Team and RStudio 2024). Read counts were calculated with summarizeOverlap (version 1.42.0, Lawrence et al. 2013) and subsequently analyzed with DeSeq2 (version 1.46.0, Love et al. 2014) to compare protoplasted and non-protoplasted samples. Finally, gene expression was normalized by transcripts per million (TPM). A volcano plot showing differentially expressed genes (DEGs) was generated with ggplot2. Genes above a log2 fold change of 3 were considered upregulated and those below −3 downregulated (Figure S4c).

#### HCR RNA-FISH

Leaf developmental zones of young, not yet unrolled leaves of 3 wk old soil-grown plants were harvested, and hairpin chain reaction RNA fluorescence in situ hybridization (HCR^TM^ RNA-FISH (v3.0), Molecular Instruments, Inc., Los Angeles, United States; Dirks and Pierce 2004; Choi et al. 2018) was conducted as described in (Spiegelhalder et al. 2024). Two fluorophores were used, 1 with signal in the green range (B1 amplifier with fluorophore 488) and 1 with signal in the red range (B2 amplifier with fluorophore 594). For each gene, between 3 and 20 probes were designed by Molecular Instruments, and 1 color fluorophore was assigned (color in the images depicted in this study corresponds to the respective fluorophore). For ease of reference, the protocol is included again here: The protocol used here was adapted from (Huang et al. 2023; Chelysheva et al. 2024) and spans 5 d from material collection to final confocal imaging. On day 1, young, not yet unrolled, leaves were collected and the bottom 5–8 mm was harvested in a Petri dish in a few drops of a fixative solution (Fixative FAA; formaldehyde solution [final concentration 4% (v/v)] (Sigma-Aldrich), glacial acetic acid [final concentration 5% (v/v)] (Thermo Fisher Scientific), absolute ethanol [final concentration 50% (v/v)] in nuclease-free water (Thermo Fisher Scientific). The leaf piece was cut once in the longitudinal and once in the transverse direction, and then collected in a 2 ml tube with Fixative FAA, and this was repeated for 4 to 5 additional leaves that were added to the same sample tube. After sample collection, a vacuum was applied to the tube several times until all leaf pieces were submerged, and then incubated at room temperature (RT) for 3 h. Fixative FAA was replaced by a series of ethanol concentrations (10%, 30%, 50%, 70%), and, for each concentration, the samples were microwaved 5 times for 30 s at 180 W (Chelysheva et al. 2024). After the last microwaving step with 70% ethanol, the sample was stored in a −20 °C freezer (the sample can be stored like this for several weeks).

On day 2, the sample was allowed to warm up to RT and then rehydrated through a series of washes at RT on a tube revolver (Thermo Fisher Scientific): first with 50% ethanol/50% DPBS-T (Tween20 [final concentration 0.1% (v/v)] (Sigma-Aldrich) in Dulbecco's phosphate-buffered saline (DPBS) (Gibco, Thermo Fisher Scientific, Massachusetts, United States) for 15 min and then twice with 100% DPBS-T for 15 min each step. Aliquots of Proteinase K solution [1 M Tris-HCl, pH 8 (final concentration 0.1 M), 0.5 M EDTA, pH 8 (final concentration 0.05 M), Proteinase K (final concentration 80 μg/ml) (Thermo Fisher Scientific) in nuclease-free water] had been prepared and frozen at −20 °C and 1 aliquot was taken out to thaw at RT during the first incubation step with 100% DPBS-T. The DPBS-T was replaced by the Proteinase K solution and incubated by applying vacuum for 5 min and then digesting for 25 min at 37 °C on the thermomixer (Eppendorf). During this step, the sample was agitated every 5 to 10 min. Subsequently, the sample was washed twice for 15 min in DPBS-T at RT on the tube rotator (Eppendorf). The second fixative solution (Fixative II; formaldehyde solution [final concentration 4% (v/v)] in DPBS-T) replaced the DPBS-T, and the sample was again vacuum-infiltrated for 10 min followed by 20 min at RT on the tube rotator. In the meantime, two 500 μl aliquots of the HCR^TM^ Probe Hybridization Buffer (Molecular Instruments) were prepared: one was left to reach RT, and the other was put at 37 °C in the thermomixer. The sample was washed twice for 15 min in DPBS-T at RT on the tube rotator and then incubated in the RT aliquot of the Probe Hybridization Buffer by applying vacuum for 10 min and then pre- hybridized for 1 h at 37 °C in the thermomixer with shaking (1,000 rpm). The probe solution was prepared by adding 0.8 pmol (ie 2 μl of the 1 μM stock) of each HCR^TM^ probe to 500 μl of the Probe Hybridization Buffer at 37 °C. For multiplexing of probes, 2 probes that can be linked to different amplifiers were added to the same tube. As much as possible was removed from the pre-hybridization solution while being careful not to pick up the leaf pieces, and the final probe solution was added to the sample before an overnight (22 h) incubation step at 37 °C in the thermomixer with shaking (1,000 rpm).

To prepare for day 3, an aliquot of HCR^TM^ Probe Wash Buffer (Molecular Instruments) was warmed up to 37 °C. Additionally, 2 aliquots of HCR^TM^ Amplification Buffer were prepared and warmed up to RT: one with 250 μl and one with 500 μl. The excess probes were washed out by washing 4 times with 500 μl Probe Wash Buffer for 15 min at 37 °C in the thermomixer with shaking (1,000 rpm). Another 2 washing steps with SSC-T buffer (20 × SSC buffer [final concentration 25% (v/v)] (Invitrogen, Thermo Fisher Scientific), Tween20 [final concentration 0.1% (v/v)] in nuclease-free water) for 5 min each at RT in the thermomixer with shaking (1,000 rpm) were carried out and the rest of the SSC-T buffer was stored at 4 °C. Next, the hairpins h1 and h2 for the amplifier B1 with fluorophore 488 and amplifier B2 with fluorophore 594 (Molecular Instruments) were put in an ice bucket to slowly thaw. In the meantime, the SSC-T buffer was replaced by 500 μl Amplification Buffer, and a vacuum was applied for 10 min before 50 min pre-amplification at RT on the tube rotator. During this incubation step, 6 pmol of hairpins h1 and h2 (ie 5 μl of the 3 μM stocks) were put in separate tubes (2 tubes for each amplifier), heated at 95 °C for 90 s, and then kept in a dark drawer at RT for 30 min. Then all hairpins were assembled in the prepared tube of 250 μl Amplification Buffer. As much as possible of the pre-amplification solution was removed while trying to avoid damage to the leaf pieces, and then the Amplification Buffer with hairpins h1 and h2 was added to the sample and incubated for about 42 h in the dark at RT.

On day 5, the SSC-T buffer was taken from the 4 °C fridge to warm up to RT. Excess hairpins were removed from the sample by washing with SSC-T buffer in a series of washes: twice for 5 min, twice for 30 min, and once for 5 min. To visualize cell walls, the sample was stained with SCRI Renaissance Stain 2200 (SR2200, Tokyo Future Style, Inc., Tsukuba City, Japan, [final concentration 0.001% (v/v)] in SSC-T buffer) or Calcofluor White (Sigma-Aldrich, [final concentration 0.0001% (w/v)] in SSC-T buffer) for 1 min and then washed 3 times with SSC-T buffer. For the imaging, a Leica Stellaris 5 (Leica Microsystems) was used with the following settings: SR2200 was excited at 405 nm excitation, green probe signal (fluorophore 488) was excited at 490 nm, red probe signal (fluorophore 594) was excited at 586 nm, and all emissions were captured within the range of the respective fluorophore emission peaks.

Subsequently, color channels were merged and adjusted for brightness and contrast in Fiji (Schindelin et al. 2012).

#### Cloning of *B. distachyon* transcriptional reporter constructs

Transcriptional reporter constructs were generated using the Greengate cloning system (Lampropoulos et al. 2013). All promoter sequences were amplified from genomic DNA (CTAB extraction method, Allen et al. 2006) of wild type (WT) *B. distachyon* Bd21-3 using the Q5 polymerase (New England Biolabs (NEB), Ipswich, United States). Promoter sequences were selected upstream of the coding sequences annotated in the *B. distachyon* Bd21-3 v1.2 genome (https://phytozome-next.jgi.doe.gov/info/BdistachyonBd21_3_v1_2). The length of promoters depended on the distance to the next gene upstream of the gene of interest and was also adjusted slightly to optimize primers. The *BdHDG2-like* promoter (*BdHDG2-likep*) was 3,584 bp and amplified using the primer pair pri121/pri122. For *BdFAMA*, the promoter (*BdFAMAp*) was 4,472 bp long and amplified with primers pri454/pri475. The *BdCST1* promoter (*BdCST1p*) was amplified in 2 fragments using the primers pri117/pri289 (fragment 1) and pri288/pri118 (fragment 2) to remove a BsaI site in the promoter. After ligating the 2 amplicons, the final amplicon was lacking a piece of the UTR, which was added by amplifying the entry module with pri820/819 and using insertional site-directed mutagenesis with the missing sequence attached to the forward primer (pri820) to complete the entry module, leading to a final promoter length of 3,555 bp. For *DUF567*, the promoter (*DUF567p*) was 1,480 bp and amplified using primers pri948/pri949. For the B module containing mCitrine with a linker, the mCitrine-linker sequence was amplified from *BdFAMAp:mCitrine-BdFAMA* (McKown et al. 2023) using the primers pri362/363. Amplicons were PCR-purified using the NucleoSpin Gel and PCR Clean-up kit (Macherey-Nagel AG, Oensingen, Schweiz) and digested with BsaI (NEB) or its isoschizomer Eco31I (Thermo Fisher Scientific). The pGGA000 (backbone for promoters) and pGGB000 (backbone for mCitrine-linker) entry vectors were digested with BsaI/Eco31I and dephosphorylated using Antarctic Phosphatase (NEB). Then, the respective inserts were ligated into the respective entry vector using the T4 ligase (Thermo Fisher Scientific) overnight at 16 °C, creating pGGA_BdHDG2-likep, pGGA_BdFAMAp, pGGA_BdCST1p, pGGA_DUF567, and pGGB_mCitrine-linker plasmids. Plasmids were transformed into *E. coli* and after overnight incubation, a colony PCR with pri30/pri47 was conducted to confirm insert lengths. Positive colonies were grown overnight to amplify the plasmid before extraction using the NucleoSpin Plasmid kit (Macherey-Nagel AG) and final sequencing to confirm the correct plasmid.

The *PvUbi2p*-driven hygromycin resistance cassette was amplified from pTRANS_250d (Čermák et al. 2017) and used to create a Greengate F module as described in (Zhang et al. 2022).

Further Greengate entry modules pGGB_3xNLS (3xNLS), pGGC012 (eGFP^NLS^), pGGC014 (eGFP) were generously provided by Prof. Dr. Karin Schumacher's group, and pGGD002 (D-dummy) and pGGE001 (rbcS terminator) were provided by Prof. Dr. Jan Lohmann's group (Lampropoulos et al. 2013).

The final transcriptional reporter constructs were created using the GreenGate protocol as described in (Lampropoulos et al. 2013). For ease of reference, the protocol is included again here: The GreenGate entry modules (pGGA_specific-promoter; pGGB_3xNLS or pGGB_mCitrine-linker; pGGC_eGFP or pGGC_eGFP-NLS; pGGD_dummy; pGGE_rbcS-terminator and pGGF_hygromycin-resistance) were repeatedly digested and ligated into the pGGZ004 (Lupanga et al. 2020) for 50 cycles (each cycle 5 min at 37 °C for digestion and 5 min at 16 °C for ligation), followed by enzyme inactivation steps at 50 °C (5 min) and 80 °C (5 min). The final ligations were transformed into *E. coli* for selection and amplification and verified by enzymatic digestion profiles. Correct assembly of the various inserts was further confirmed by sequencing (whole-plasmid sequencing or Sanger sequencing of the overhangs).

#### Design and cloning of CRISPR constructs

CRISPR guides were selected using the “Find CRISPR sites” tool of Geneious Prime (version 2024.0.3, GraphPad Software LLC d.b.a Geneious, activity scoring based on (Doench et al. 2016)). The selected guides and their reverse complement with added cloning overhangs for *BdGRAS32* (pri1457 and pri1458) and *BdPME53-like* (pri887 and pri888) were annealed and phosphorylated using the T4 PNK (NEB). The JD633 plasmid (Debernardi et al. 2020) was used as a CRISPR backbone and first digested using the PaqCI enzyme (NEB), followed by dephosphorylation using Antarctic Phosphatase (NEB). The annealed guides and the backbone were ligated overnight at 16 °C using the T4 DNA ligase (NEB). Competent *E. coli* NEB 5-alpha (NEB) were transformed with the CRISPR plasmids and amplified overnight. The next day, colony PCR with a primer binding on the CRISPR backbone (pri92) and the respective reverse guide was conducted to identify positive colonies, which were then grown overnight to amplify the plasmid. Plasmids were isolated with the NucleoSpin Plasmid kit (Macherey-Nagel AG) and sent for sequencing using pri92 to check for correct insertion of the guide.

#### *B. distachyon* plant transformation

To prepare the constructs for transformation, *A. tumefaciens* AGL1 (Bragg et al. 2012) was transformed with the respective plasmids and grown on plates overnight at 28 °C. Positive colonies were used for the transformation of embryonic calli of wild type *B. distachyon* Bd21-3 following the plant transformation protocol described in (Zhang et al. 2022). For ease of reference, the protocol is included again here: Young, transparent embryos were isolated and grown for 3 wk at 28 °C in the dark on callus induction media (CIM; per L: 4.43 g Linsmaier & Skoog basal media (LS; Duchefa), 30 g sucrose, 600 ml CuSO_4_ (1 mg/ml, Sigma/Merck), 500 ml 2,4-D (5 mg/ml in 1 M KOH, Sigma/Merck), pH 5.8, plus 2.1 g of Phytagel (Sigma/Merck). After incubation, crisp, yellow callus pieces were subcultured to fresh CIM plates and further incubated at 28 °C in the dark for 2 wk. Then, calli were broken down to 2-5 mm small pieces, transferred to fresh CIM plates, and subcultured for 1 more week at 28 °C in the dark. For transformation, agrobacteria with the desired construct were dissolved in liquid CIM media (same media as above, without the phytagel) with freshly added 2,4-D (2.5 mg/ml final concentration), Acetosyringone (200 mM final concentration, Sigma/Merck), and Synperonic PE/F68 (0.1% final concentration, Sigma/Merck). The OD600 of the agrobacteria solution was adjusted to 0.6. Around 100 calli were incubated for at least 10 min in the bacteria solution, dried off on sterile filter paper, and incubated for 3 d at room temperature in the dark. After incubation, transformed calli were moved to selection media (CIM + Hygromycin (40 mg/ml final concentration, Roche, Basel, Switzerland) + Timentin (200 mg/ml final concentration, Ticarcillin 2Na & Clavulanate Potassium (Duchefa)) and incubated for 1 wk at 28 °C in the dark. Then, calli were moved to fresh selection plates and incubated for 2 more weeks at 28 °C in the dark. Next, calli were moved to callus regeneration media (CRM; per L: 4.43 g of LS, 30 g maltose (Sigma/Merck), 600 ml CuSO4 (1 mg/ml), pH 5.8, plus 2.1 g of Phytagel). After autoclaving, cool down and add Timentin (200 mg/ml final concentration), Hygromycin (40 mg/ml final concentration), and sterile Kinetin solution (0.2 mg/ml final concentration, Sigma/Merck). Calli were incubated at 28 °C with a 16 h light:8 h dark cycle (70–80 mmol PAR m^−2^ s^−1^). After 1–6 wk in the light, shoots will form. Shoots that are longer than 1 cm and ideally have 2 or more leaves were moved to glass cups for rooting (Weck glass and packaging GmbH, Bonn, Germany) containing rooting media (per L: 4.3 g Murashige & Skoog including vitamins (Duchefa), 30 g sucrose, adjust pH to 5.8, add 2.1 g Phytagel. After autoclaving, cool down and add Timentin (200 mg/ml final concentration). Once roots had formed, plantlets were moved to soil (consisting of 4 parts Einheitserde CL ED73 (Einheitserdewerke, Werkverband e.V., Sinntal-Altengronau, Germany) and 1 part Vermiculite) and grown in a greenhouse or growth chamber with 18 h light:6 h dark cycle (250–350 mmol PAR m^−2^ s^−1^). For the first 2–3 d, trays with the transgenic plantlets would still be covered with a transparent lid to maintain humidity.

#### Imaging of abaxial mature epidermis

To obtain the image of the abaxial leaf epidermis in Fig. 2a, lignin of a mature leaf was stained with Basic Fuchsin (Sigma-Aldrich) and imaged together with cell wall autofluorescence as described in (Nunes et al. 2023). For ease of reference, the protocol is included again here: Small leaf fragments previously fixed and cleared in 7:1 ethanol:acetic acid were washed in 70% ethanol and then transferred to distilled water with 0.02% (v/v) Tween20 (Sigma-Aldrich) for rehydration for 3 h. Samples were incubated in 30 ml of 0.01% Basic Fuchsin for 5 min and washed twice with 30 ml of 50% (v/v) glycerol for 5 min (2.5 min per washing step) and then mounted in 50% (v/v) glycerol for imaging. Samples were imaged on a Stellaris 5 confocal microscope (Leica Microsystems). Cell wall autofluorescence was excited at 405 nm and emissions captured in the range of 490–550 nm. Basic Fuchsin was excited at 561 nm, and emissions were captured between 573–603 nm. Stacks of 0.33 µm steps were obtained. For the image in Fig. 2a, a sum of slices Z-projection was performed in Fiji (Schindelin et al. 2012).

#### Imaging of reporter lines

For *DUF567p:mCitrine-eGFP^NLS^* and *BdFAMAp:mCitrine-eGFP^NLS^*, the developmental zone of the youngest, not yet unrolled leaf of 2–3 wk old plants was imaged. For *BdHDG2-likep:^3xNLS^eGFP* and *BdCST1p:mCitrine-eGFP^NLS^*, second leaves of plate-grown seedlings were imaged 5–7 d after germination. Cell walls were stained with propidium iodide (PI (Invitrogen, Thermo Fisher Scientific), 0.5% (v/v)) for 3–5 min. A Leica Stellaris 5 confocal microscope (Leica Microsystems) with the following settings was used to visualize the different channels: PI was excited at 549 or 584 nm, mCitrine was excited at 515 nm, and eGFP was excited at 489 nm. Emissions were captured in the range of the emission peak of the respective fluorophore. Subsequently, color channels were merged and adjusted for brightness and contrast in Fiji (Schindelin et al. 2012). For the *BdCST1* reporter, z-stacks were taken, and Maximum Projection images were created.

Imaging of the barley reporter line *HvFCP1p:VENUS-H2B* (Vardanega et al. 2025) was performed with the confocal microscope Zeiss LSM880 (Carl Zeiss AG, Oberkochen, Germany), with Plan-Apochromat 20×/0.8 or Plan-Apochromat 40x/1 objectives. Fresh barley shoot apical meristems and leaf primordia were stuck on a double-sided adhesive tape on an objective slide, stained with PI (0.3 mM) for 3 min, washed 3 times with water, and subsequently covered with a cover slide before being placed under the microscope. PI was excited at 561 nm and Venus at 514 nm. Signal was collected within the fluorophore's emission peak. Pictures were analyzed using Fiji (Schindelin et al. 2012).

#### *Hordeum vulgare* multiplex smRNA-FISH

smRNA-FISH (Molecular Cartography) of the *Hordeum vulgare* (barley) cultivar Golden Promise was conducted as described in the associated publication (Demesa-Arevalo et al. 2026). For ease of reference, the protocol is included again here: Vegetative shoot apical meristems and surrounding leaf primordia were fixed overnight in phosphate-buffered saline (PBS) containing 4% paraformaldehyde (PFA) and 0.03% Triton X-100 (Sigma-Aldrich), dehydrated through an ethanol series, and embedded under vacuum in Paraplast Extra (Leica Biosystems, Deer Park, United States). 10 μm sections of the embedded tissues were placed on glass slides (Resolve Biosciences), deparaffinized, gradually rehydrated, and permeabilized with proteinase K (Thermo Fisher Scientific). After re-fixation, acetylation, and dehydration through an ethanol series, the slides were mounted with SlowFade-Gold Antifade Mountant (Invitrogen, Thermo Fisher Scientific). Probe hybridization and imaging were performed by Resolve Biosciences. Image analysis was performed in Fiji (Schindelin et al. 2012) using the Polylux plugin (Polylux_V1.9.0) from Resolve Biosciences.

#### Genotyping of CRISPR mutant lines

To genotype the CRISPR mutant lines, genomic DNA was extracted from T0 and T1 transgenic plants using the CTAB extraction method (Murray and Thompson 1980). Genotyping primers were selected up- and downstream of the CRISPR guide site to amplify the putative mutation site (*bdgras32*: pri1627 and pri1621, *bdpme53-like*: pri889 and pri890). The mutation site was PCR-amplified, and the correct size of the amplicon was confirmed in a standard 1% agarose gel. After PCR purification with the NucleoSpin Gel and PCR Clean-up kit (Macherey-Nagel AG), the PCR product was sent for sequencing with the added primer pri1621 (for *bdgras32*) or pri889 (for *bdpme53-like*) to identify or confirm the mutation and the zygosity.

#### Phenotyping of the *bdgras32* mutant line

6 d after germination, the second leaves of plate-grown seedlings of wild type (WT) and *bdgras32* (T1 generation) were imaged on a Stellaris 5 confocal microscope (Leica Microsystems). Cell walls were stained using propidium iodide (PI, 0.5% (v/v)) for 4 min. PI was excited at 549 nm, and emissions were captured around the emission peak. Images were taken of cell files around developmental stage 6 to show the misdivision phenotype, and around the first asymmetric epidermal divisions (ie, developmental stage 1 to 2) for subsequent analysis. Stitching of images was done in Fiji (Schindelin et al. 2012) using the pairwise stitching tool (Preibisch et al. 2009) and Paint.NET (version 5.1.9, https://www.getpaint.net/index.html). Then, stitched sequences were cropped to frames of the same length (388.93 µm) with the first asymmetric divisions approximately in the center (see Fig. 7h). 2D segmentation of 5 cell files including 1 stomatal lineage cell file was conducted in MorphoGraphX (version 2.0.1-379, Barbier de Reuille et al. 2015; Strauss et al. 2021, 2022) as follows: Images were adjusted with Brighten/Darken (value = 3) and the signal was projected onto the mesh (minimum distance 0, maximum distance 2) before auto segmentation. Mistakes were corrected by manual tracing and watershed segmentation until a correct cell mesh was created. Only cells were kept that are fully visible in the frame. Corner triangles were fixed, the mesh was smoothened (walls only, 3 passes), and then Heatmap Classic (manual range 0–300) was used to calculate and visualize cell areas (Fig. 7h).

For fresh and dry weight measurements, above-ground tissue of plants was harvested 5 wk after germination by cutting and collecting the whole plant 1 cm above soil level. Fresh weight was measured immediately after cutting (Excellence Plus XP, Mettler-Toledo, Greifensee, Switzerland). Each individual was kept in a seed bag for 3 d at 65 °C and then transferred to a closed container with silica beads for 1 d to finalize the drying process. Afterwards, the dried plants were weighed to receive dry weight values.

Statistical significance analyses using an unpaired, two-sided Student's *t* test (stats package version 4.3.1, R Core Team and R Foundation for Statistical Computing 2025) and visualization of results were done in R. Details of the statistical testing are in Table S8. The R script for mutant analysis, as well as the data table output of MorphoGraphX, is available on GitHub: https://github.com/raissig-lindner-lab/Berg-et-al_2025_ScSeq.

#### Phenotyping of *the BDPME53-like* mutant line

Leaf-level gas exchange measurements of wild type (WT) and *bdpme53-like* (T1 generation) plants were conducted and subsequently analyzed as described in (Berg et al. 2025). For ease of reference, the protocol is included again here: Physiological parameters for each genotype were obtained from 3–4 wk old soil-grown, non-flowering plants. The youngest, fully expanded leaf was measured using the LI-6800 Portable Photosynthesis System with the 6800-01A Multiphase Flash Fluorometer chamber (LI-COR Biosciences Inc., Lincoln, NE, United States) in the 2 cm^2^ chamber. Environmental conditions were programed as described in (Nunes et al. 2022): flow rate, 500 µmol s^−1^; fan speed, 10,000 rpm, and leaf temperature, 28 °C. Gas exchange in reaction to changing light intensities was measured at a relative humidity 40% and a CO_2_ concentration of 400 µmol mol^−1^. Values were automatically logged every 60 s with 20 min steps for the light intensities in units of photosynthetically active radiation (PAR), 1,000 (high light) - 100 (low light) - 1,000 - 0 (darkness) PAR m^−2^ s^−1^. PAR is equivalent to Photosynthetic photon flux density (PPFD), which is in μmol m^−2^ s^−1^.

As *B. distachyon* leaves are not broad enough to fill the 2 cm^2^ chamber, data from each run were corrected manually in the Excel sheet by individual leaf area, which was calculated as leaf width multiplied by the chamber diameter. Subsequent averaging of values across all individuals per species, outlier removal, correction by stomatal density, and final visualization were conducted in R Studio using the licornetics R package (version 2.1.2, Berg 2024; Berg et al. 2025) that is available on GitHub with further documentation (https://github.com/lbmountain/licornetics).

Leaves were collected after gas exchange measurements and fixed in 7:1 ethanol:acetic acid for at least 24 h. Samples were rinsed in water and mounted on slides in Hoyer's solution (70% (w/v) chloralhydrate (Roth, Karlsruhe, Germany), 4% (w/v) glycerol (Thermo Fisher Scientific), 5% (w/v) gum arabic (Roth)). The abaxial leaf side was imaged using a Leica DM2000 DIC microscope (Leica Microsystems). For stomatal density, 3 fields of view (image size 0.198 mm^2^) per leaf were counted in Fiji (Schindelin et al. 2012), resulting in 29–53 stomata per individual and divided by the image size to obtain stomatal density per mm^2^. Statistical significance analysis using an unpaired, two-sided Student's *t* test (stats package version 4.3.1, R Core Team and R Foundation for Statistical Computing 2025) and visualization of results were done in R. Details of the statistical testing are in Table S8. The R script for mutant analysis is available on GitHub: https://github.com/raissig-lindner-lab/Berg-et-al_2025_ScSeq.

#### *B. distachyon* developing leaf single-cell atlas web tool

To create an expression browser, we generated a template onto which gene expression can be plotted. First, we created the template file following the tutorial provided in the ggPlantmap GitHub: https://github.com/leonardojo/ggPlantmap/blob/main/guides/TutorialforXMLfile.pdf. In short, the Icy software (version 2.5.2.0, https://icy.bioimageanalysis.org/) was used to trace and create a template with outlines and labels for different tissues and cell types, and save them as .xml. In R Studio, the ggPlantmap package (version 1.1.0, Jo and Kajala 2024) was used to convert the .xml file to a ggPlantmap object. The Seurat AverageExpression function was used to extract the mean expression of genes per cluster identity. For the main tissue assignments, the whole dataset was used and Seurat clusters were labeled for analysis as follows: Shoot apex = cluster 3, 11, 21, 22, 24, 28; vasculature = clusters 10, 17, 19, 20; mesophyll = clusters 0, 1, 2, 4, 8, 9, 14, 15; epidermis = clusters 5, 7, 12, 13, 18, 25, 26, 27, 29, 30, 31. For annotation of hair cells (HCs), silica cells, and inter-specialized cells, the epidermal subset was used, and Seurat clusters were labeled for analysis as follows: Silica cells = clusters 0, 6, 9, 21; early HCs = cluster 2; middle HCs = cluster 8; late HCs = cluster 14; inter-specialized cells = clusters 4, 7, 17. For stomatal lineage cell types and stages, the stomatal lineage subset was used, and Seurat clusters were labeled for analysis as follows: Early GMCs = cluster 9; dividing GMCs = clusters 14, 16; early GCs = cluster 7; late GCs = cluster 12; SCs = cluster 5. The ggPlantmap.heatmap function was then used to plot gene expression onto the previously created template.

The UMAP feature plots depicting gene expression were created as follows: The Seurat FetchData function was used to obtain UMAP locations of cells, their assigned identity, and the expression of genes (features) in a given cell. Visualization of the expression of a gene of interest was done using the ggplot2 package. The shiny package (version 1.9.1, Chang et al. 2024) was used to create the website, which was then deployed with Docker (Docker Inc., Palo Alto, United States). Further packages required for the creation of the web tool: tidyverse (version 2.0.0, Wickham et al. 2019), Cairo (version 1.6–2, Urbanek and Horner 2023), bslib (version 0.8.0, Sievert et al. 2024), shinythemes (version 1.2.0, Chang 2021), shinycssloaders (version 1.1.0, Attali and Sali 2024), MetBrewer (version 0.2.0, Mills 2022), and arrow (version 20.0.0.2, Richardson et al. 2025).

The R script used to create the web tool (also works locally) is available on GitHub: https://github.com/raissig-lindner-lab/Berg-et-al_2025_ScSeq.

#### Reagents and resources

Information on organisms, bacterial strains, reagents, deposited data, oligonucleotides, recombinant DNA, and software was compiled in Table S9.

### Accession numbers

Gene accession numbers and related genes in other species can be found in Table S3.

## Supplementary Material

koag172_Supplementary_Data

## Data Availability

Single-cell RNA-sequencing and bulk RNA-sequencing data have been deposited at GEO and are publicly available (GEO: GSE307277 and GSE306756). The expression browser of the single-cell RNA-sequencing dataset can be found online at https://shiny.ips.unibe.ch. All quantitative data generated and analyzed in this study can be found in the supplementary materials. Microscopy images reported in this paper will be shared by the lead contact upon request. R scripts used to analyze the datasets in this study, as well as a step-by-step protocol for protoplasting and HCR RNA-FISH of *B. distachyon* leaf tissue can be found on Github: https://github.com/raissig-lindner-lab/Berg-et-al_2025_ScSeq. Any additional information required to reanalyze the data reported in this paper is available from the lead contact upon request.
