# Supplementary material for: The developing leaf of the wild grass Brachypodium distachyon at single-cell resolution
Source: Plant Cell. 2026 Jun 10;38(6):koag172. doi: 10.1093/plcell/koag172 (PMC13291820; doi:10.1093/plcell/koag172)
Supplement: koag172_Supplementary_Data [file koag172_supplementary_data.zip › TPC-2025-1212R1_Supplementary Data.pdf]

Supplementary Information

for

Berg et al. (2026) *Plant Cell*

This file contains:

Figures S1 - S7

Tables S1 - S3

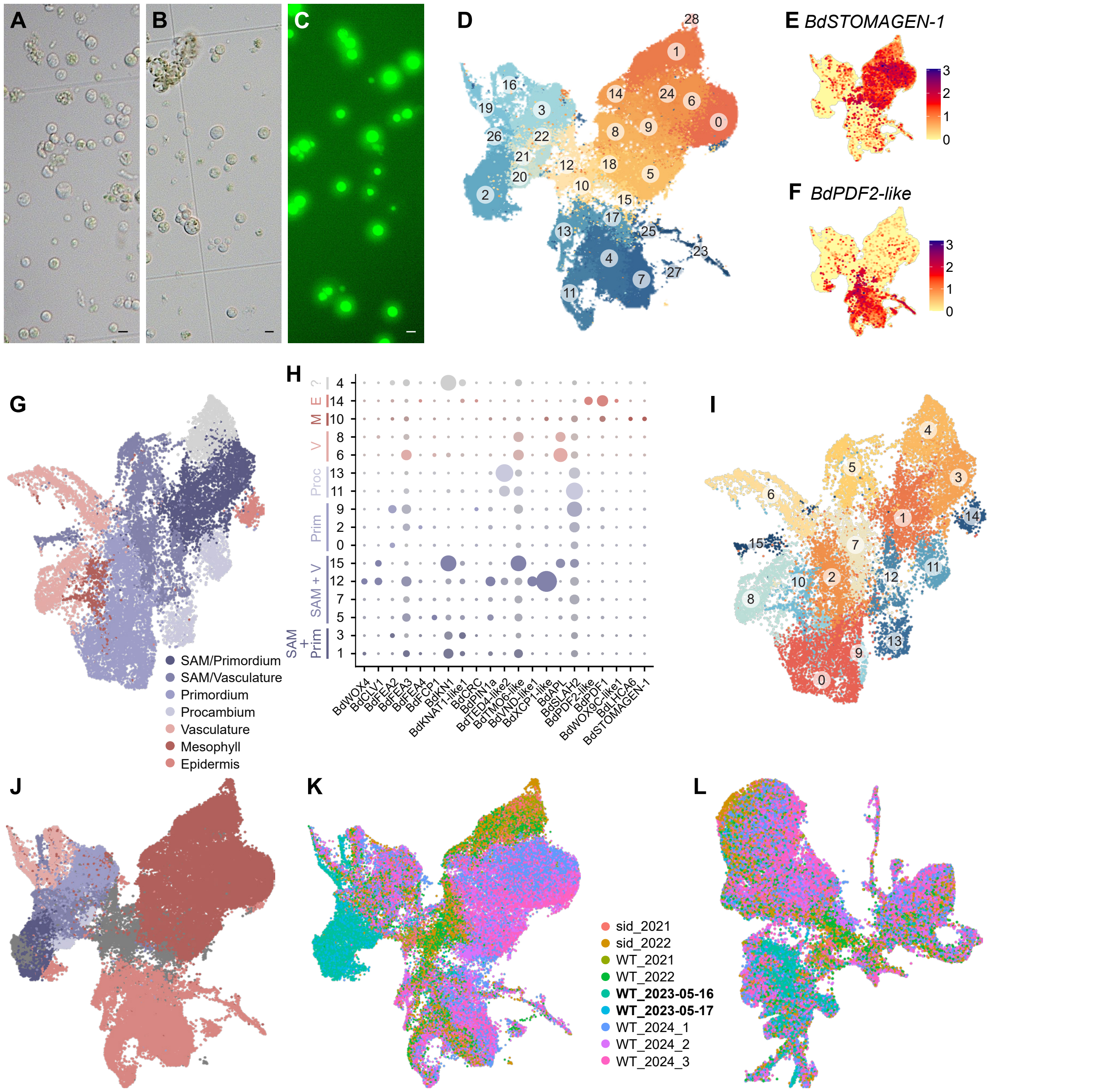

Figure S1. Semi-supervised integration of the datasets. Related to Fig. 1. **(A, B)** DIC images of protoplasted cells. Scale bars, 10 µm. **(C)** Epifluorescence microscopy image of protoplasts stained with fluorescein diacetate (FDA). Viable cells glow green. Scale bar, 10 µm. **(D)** Un-integrated whole dataset UMAP plot with color indicating Seurat clusters; n = 69,687 cells. **(E, F)** Un-integrated whole dataset UMAP feature plots of marker genes for the leaf mesophyll (E) and epidermis (F) with color indicating expression strength. **(G-I)** Re-analyzed subset containing clusters 2, 3, 16, 19, 20, 21, 22, and 26. UMAP plots with color indicating tissue type (G) or Seurat clusters (I). Dot plot in (H) shows expression of marker genes in the clusters from (I). Dot size represents the percentage of cells within a cluster that express the gene and color saturation represents expression strength. Tissues are color-coded as in (G). **(J)** UMAP plot of the un-integrated whole dataset with color indicating tissue type. Cell labels from the subset in (G) were transferred to this plot. **(K, L)** Whole dataset UMAP plot with color indicating the library origin in the un-integrated (K) and integrated (L) dataset. SAM & primordia libraries in bold.

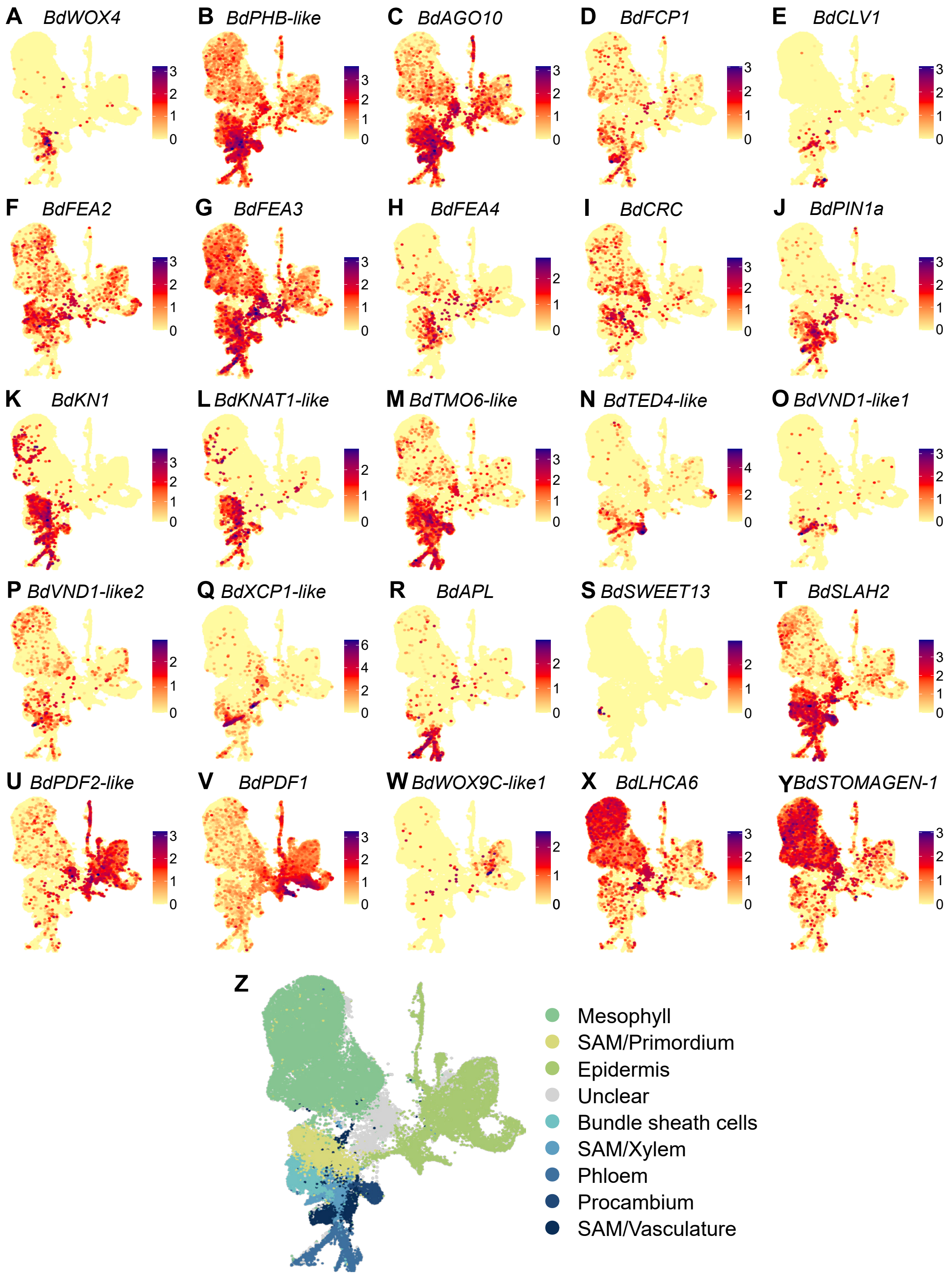

Figure S2. Feature plots of leaf tissue marker genes. Related to Fig. 1. **(A-T)** Whole dataset UMAP feature plots of marker genes for shoot apical meristem (SAM) and early vasculature. *BdKNOTTED1* (K) and *BdSLOWLY ACTIVATING ANION CHANNEL1 (SLAC1) HOMOLOGUE 2* (*BdSLAH2*, T) are also shown in Fig. 1E with a different color scheme. **(U-W)** Whole dataset UMAP feature plots of marker genes for the leaf epidermis. *BdPROTODERMAL PATTERNING FACTOR 2-like* (*BdPDF2-like*; U) is also shown in Fig. 1E with a different color scheme. **(X, Y)** Whole dataset UMAP feature plots of marker genes for the mesophyll. *BdSTOMAGEN-1* (Y) is also shown in Fig. 1E with a different color scheme. **(Z)** Whole dataset UMAP plot with colour indicating tissue and cell type assignment. Each dot in the UMAP plots represents the transcriptome of a single cell. Color legends in the UMAP feature plots indicate expression strength.

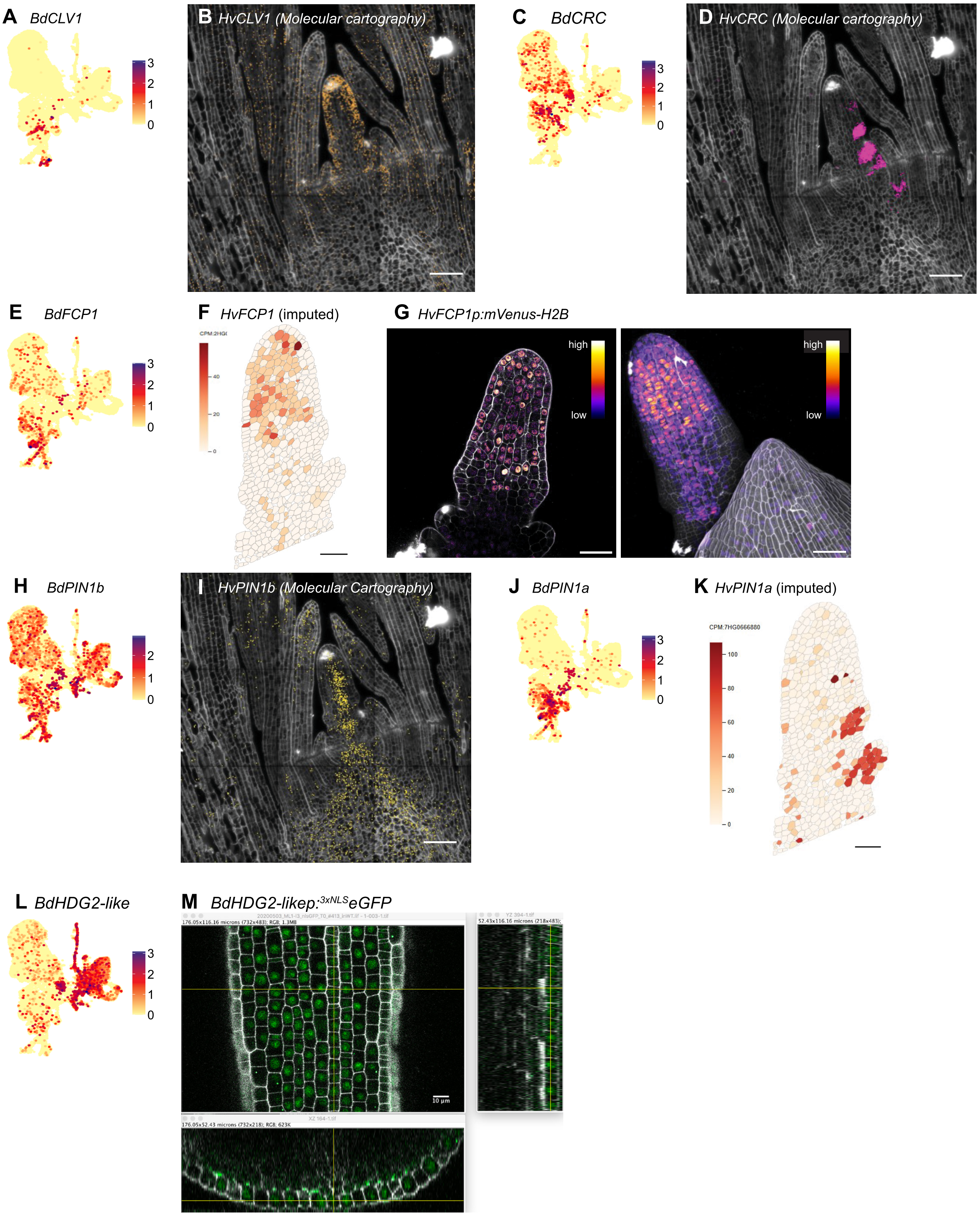

Figure S3. Meristematic marker gene expression. Related to Fig. 1. **(A)** Whole dataset UMAP feature plot of *BdCLAVATA1* (*BdCLV1*). Same plot as in Fig. S2E. **(B)** smRNA-FISH (Molecular cartography) image of barley *HvCLV1* in the shoot apex with early leaves. This image is a composite and the same background image is also used for panels D and I. Full size version of the image shown in Fig. 1c of (Vardanega et al. 2025) with different color scheme. Also shown as a zoom-in without gene expression channel in Fig. 1B of (Demesa-Arevalo et al. 2026). **(C)** Whole dataset UMAP feature plot of *BdCRABS CLAW* (*BdCRC*). **(D)** smRNA-FISH image of barley *HvCRC* in the shoot apex with early leaves. Full size version of the image shown in Fig. 3F of (Demesa-Arevalo et al. 2026) with different color scheme and only *HvCRC* expression. Also shown as a zoom-in without gene expression channel in Fig. 1b of (Demesa-Arevalo et al. 2026). **(E)** Whole dataset UMAP feature plot of *BdFON2-LIKE CLE PROTEIN 1* (*BdFCP1*). Same plot as in Fig. S2D. **(F)** Imputed expression of barley *HvFCP1*. Dataset and imputation method are described in (Demesa-Arevalo et al. 2026). **(G)** Expression of *HvFCP1p:Venus-H2B* in barley vegetative shoot apical meristem (vSAM, left) and leaf primordial epidermis (right, at Waddington stage 1-1.5). Color legend indicates expression strength. Cell walls stained with propidium iodide (PI, gray). **(H)** Whole dataset UMAP feature plot of *BdPINFORMED1b* (*BdPIN1b*). **(I)** smRNA-FISH image of barley *HvPIN1b* (also known as *HvPIN1*). Also shown as a zoom-in without gene expression channel in Fig. 1b of (Demesa-Arevalo et al. 2026). **(J)** Whole dataset UMAP feature plot of *BdPIN1a*. Same plot as in Fig. S2J. **(K)** Imputed expression of barley *HvPIN1a*. Dataset and imputation method are described in (Demesa-Arevalo et al. 2026). **(L)** Whole dataset UMAP feature plot of *BdHOMEODOMAIN GLABROUS2-like* (*BdHDG2-like*). **(M)** Expression of *BdHDG2-likep:^{3xNLS}eGFP* in *B. distachyon* leaf epidermis (z-stack) with orthogonal sections indicated by yellow lines; transverse section below, longitudinal section to the right. [...legend continued on next page...]

Figure S3 (continued). Color in the smRNA-FISH images indicates expression of the gene of interest. Each dot in the UMAP plots represents the transcriptome of a single cell. Color legends in the UMAP feature plots and imputed data indicate expression strength. Scale bars, 50 μm, unless otherwise indicated.

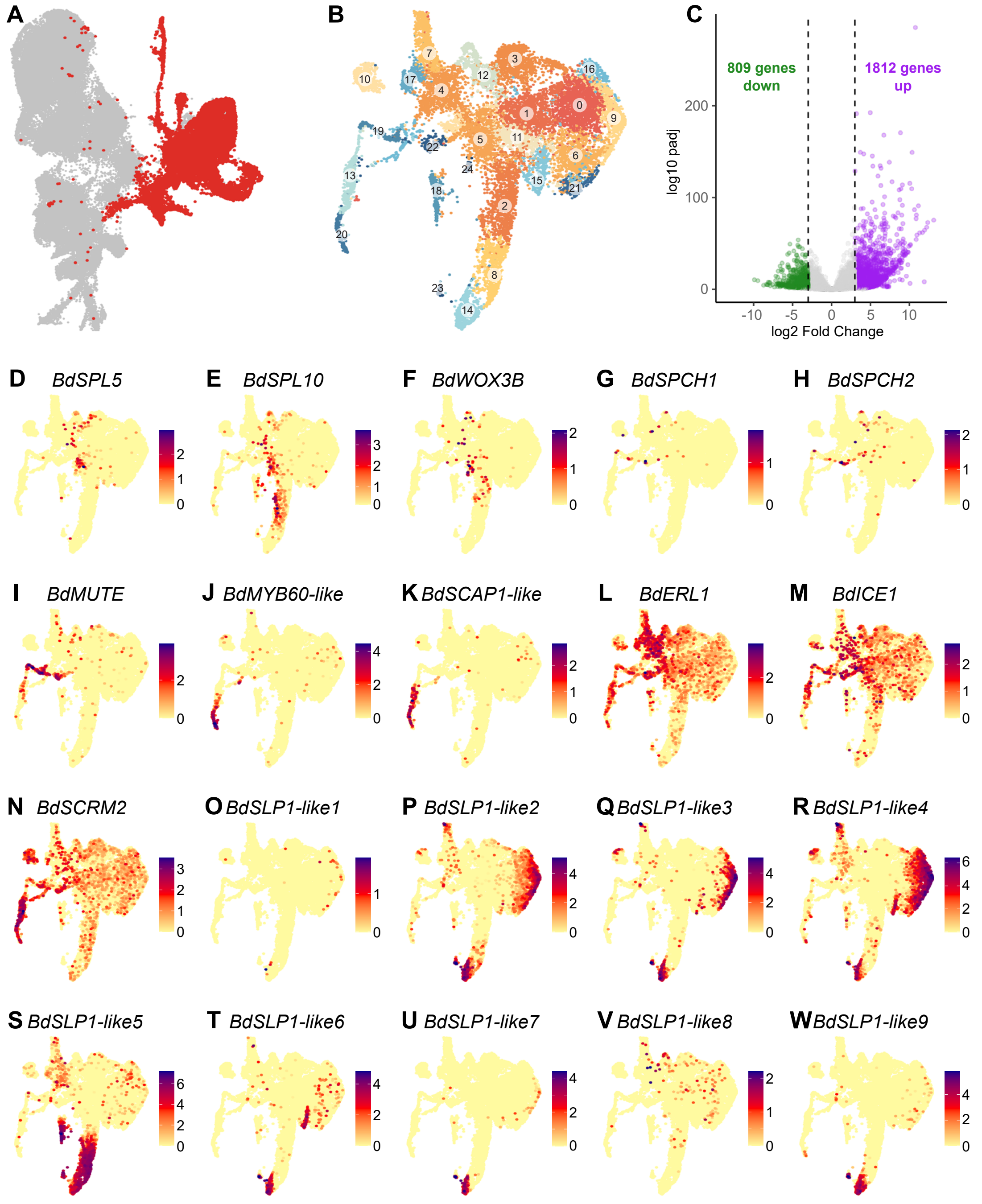

Figure S4. Feature plots of epidermal marker genes. Related to Fig. 2. **(A)** Whole dataset UMAP plot with cells belonging to the epidermal subset highlighted in red. **(B)** UMAP plot of the epidermal subset with numbered Seurat clusters; n = 15,034 cells. **(C)** Volcano plot showing logarithmic fold change of genes in response to the protoplasting protocol. Genes are considered downregulated (green) if the log2fold change is < -3 and upregulated (magenta) if the fold change is > 3. **(D-F)** Epidermis UMAP feature plots of hair cell lineage marker genes. **(G-N)** Epidermis UMAP feature plots of stomatal lineage marker genes. **(O-W)** Epidermis UMAP feature plots of the *BdSILIPLANT1-like* (*BdSLP1-like*) family. *BdSLP1-like3* (Q) and *BdSLP1-like5* (S) are also shown in Fig. 2L,M. *BdSLP1-like1* (O) is protoplasting-affected (Table S5). Each dot in the UMAP plots represents the transcriptome of a single cell. Color legends in the UMAP feature plots indicate expression strength.

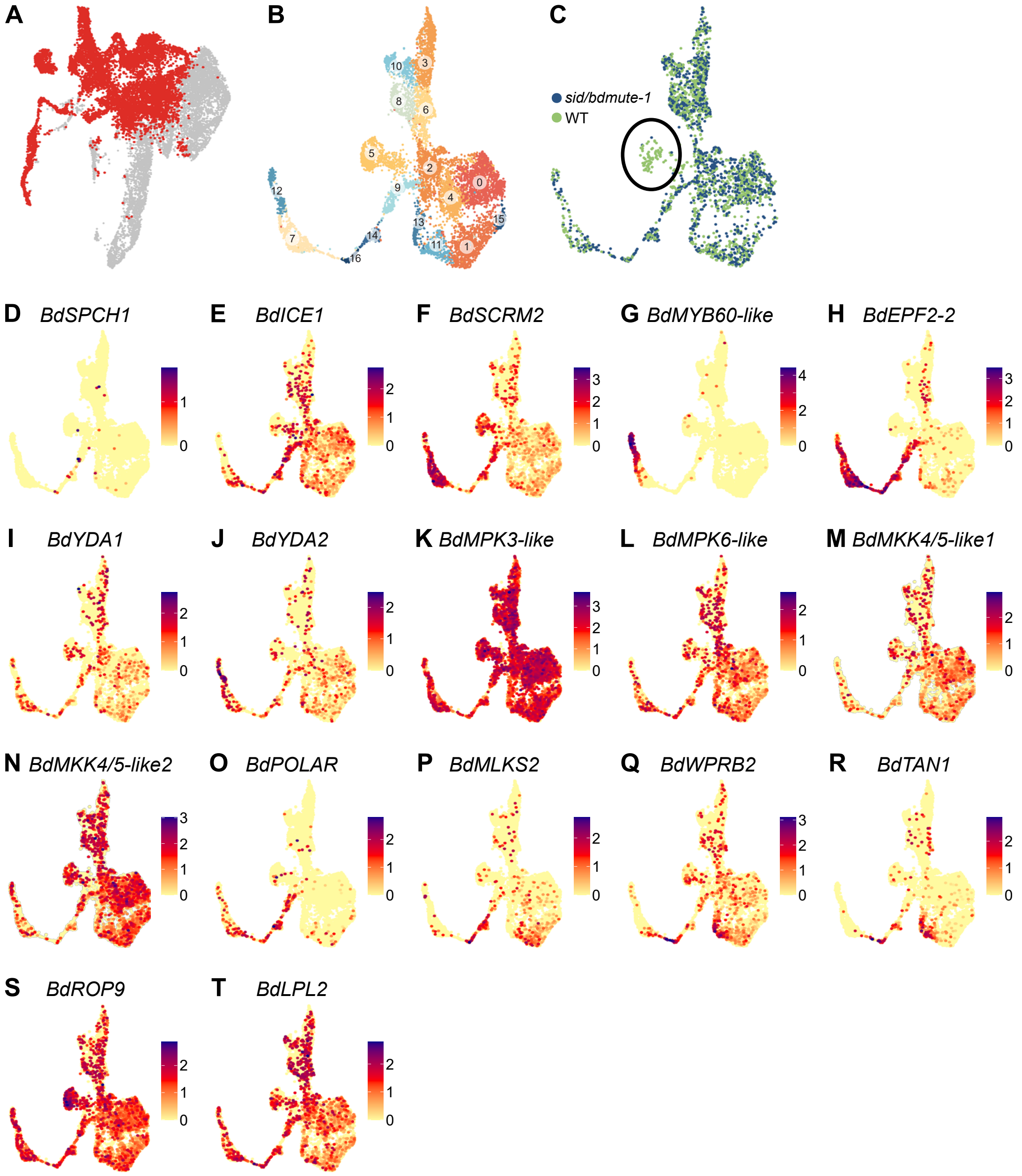

Figure S5. Feature plots of stomatal lineage marker genes. Related to Fig. 3 and 5. **(A)** Epidermis UMAP plot with cells belonging to the stomatal lineage subset highlighted in red. **(B)** UMAP plot of the stomatal lineage subset with numbered Seurat clusters. **(C)** Stomatal lineage UMAP plot with color indicating genotype. Same UMAP plot as in (B), but only the two wild-type and two *sid/bdmute-1* libraries generated at the same time are shown (i.e., the two 2021 and the two 2022 datasets, Fig. S1L). The subsidiary cell (SC) cluster enriched for wild type (WT) cells is indicated by a circle. **(D-H)** Stomatal lineage UMAP feature plots of stomatal lineage marker genes. **(I-N)** Stomatal lineage UMAP feature plots of *MITOGEN-ACTIVATED PROTEIN* (*MAP*) kinase genes potentially involved in stomatal development. **(O-T)** Stomatal lineage UMAP feature plots of genes involved in the subsidiary mother cell (SMC) division of grasses. Each dot in the UMAP plots represents the transcriptome of a single cell. Color legends in the UMAP feature plots indicate expression strength.

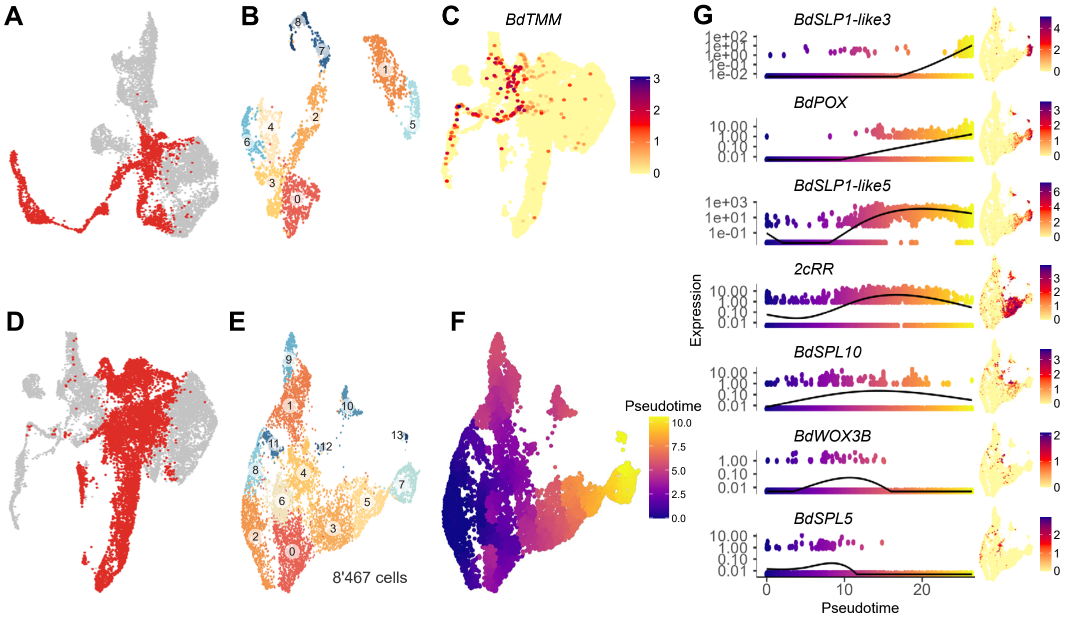

Figure S6. Pseudotime analysis of the stomatal and hair cell lineage. Related to Fig. 4. **(A)** Stomatal lineage UMAP plot of the stomatal lineage subset with cells belonging to the guard cell (GC) lineage subset highlighted in red. **(B)** UMAP plot of the GC lineage subset with numbered Seurat clusters. **(C)** Epidermis UMAP feature plot of *BdTOO MANY MOUTHS* (*BdTMM*). **(D)** UMAP plot of the epidermal subset with cells belonging to the hair cell (HC) lineage highlighted in red. **(E)** HC lineage UMAP plot with numbered Seurat clusters. N = 8’467 cells. **(F)** UMAP plot of the HC lineage subset with color indicating pseudotime. **(G)** Dot plot showing expression of marker genes across the HC lineage subset pseudotime gradient. UMAP feature plots of the respective genes are shown on the right. Color legends in the UMAP feature plots indicate expression strength.

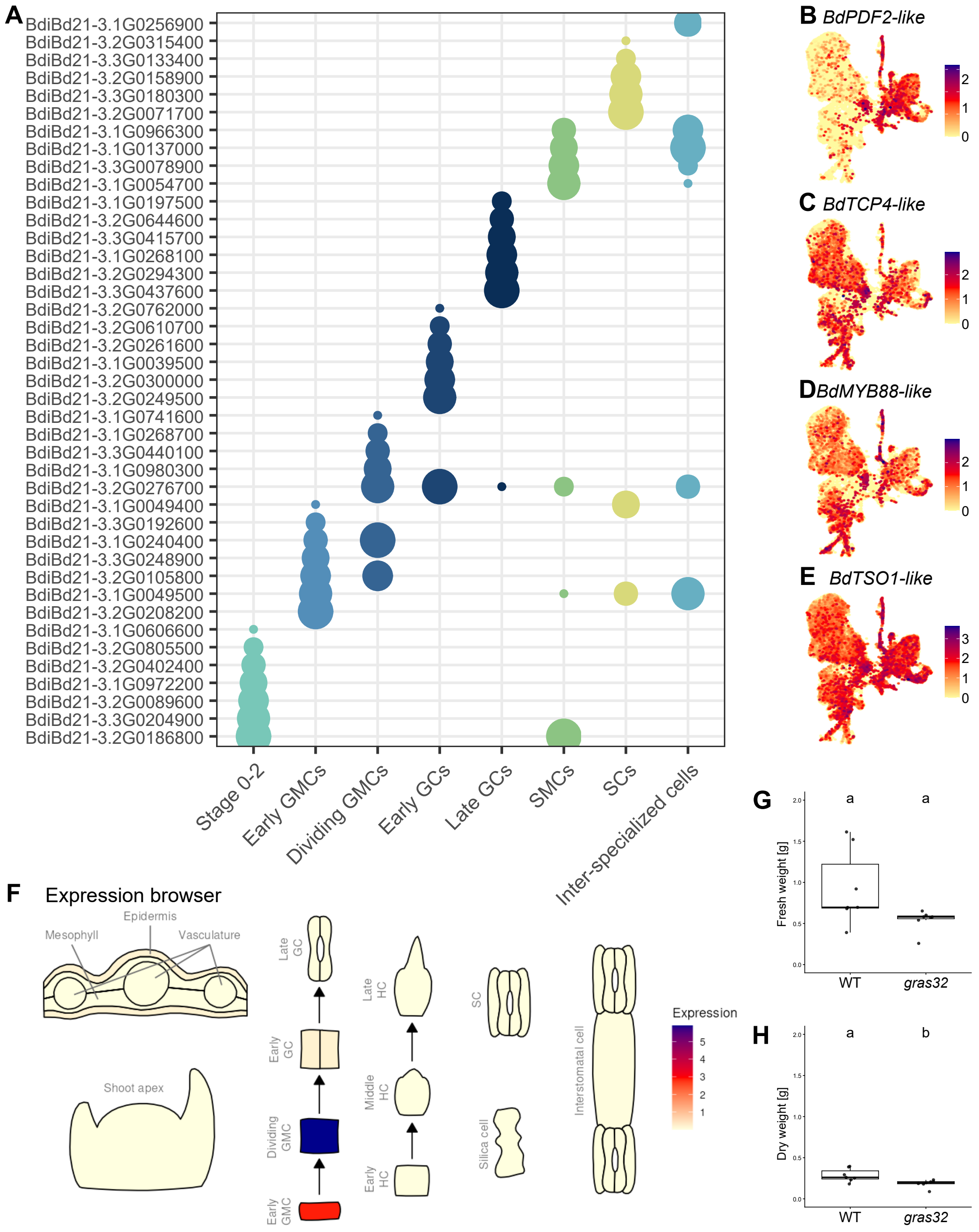

Figure S7. Stomatal regulons and single-cell gene expression browser. Related to Fig. 6 and Fig. 7. **(A)** Dot plot showing the top 7 transcription factors (TFs)/regulons per cluster. Same plot as in Fig. 6A, here with *B. distachyon* Bd21-3 gene accession numbers instead of names. **(B)** Whole dataset UMAP feature plot of *BdPROTODERMAL PATTERNING FACTOR 2-like* (*BdPDF2-like*). Same plot as in Fig. S2U and also shown in Fig. 1E, there with a different color scheme. **(C)** Whole dataset UMAP feature plot of *BdTEOSINTE BRANCHED 1, CYCLOIDEA, PROLIFERATING CELL FACTOR1/2 4-like* (*BdTCP4-like*). **(D)** Whole dataset UMAP feature plot of *BdMYB88-like*. **(E)** Whole dataset UMAP feature plot of *BdTSO1-like*. **(F)** Representative image of the expression overview map which is accessible for any *B. distachyon* Bd21-3 gene on the website introduced in this study (here shown for *BdMUTE*). **(G, H)** Fresh (G) and dry (H) weight measurements of aboveground tissue of wild type (WT) and *bdgras32* (*gras32*) plants; n = 7 individuals per genotype. Each dot indicates one individual. Significant differences calculated with unpaired, two-sided Student's *t*-test ($p<0.5$) are indicated by differing letters. Each dot in the UMAP plots represents the transcriptome of a single cell. Color legends in the UMAP feature plots indicate expression strength.

Table S1. General overview of the scSeq datasets.

Dataset	Tissue	Location	Nr. of cells after filtering	Mean/median nr. of UMI	Mean/median nr. of features	GEO identifier
WT 2021	Leaf develop-mental zone	Heidelberg (DE), Green-house	2’656	12’929/11’594	3’674/3’894	GSM9220335
sid 2021	Leaf develop-mental zone	Heidelberg (DE), Green-house	3’427	10’256/8’515	3’189/3’186	GSM9220336
WT 2022	Leaf develop-mental zone	Heidelberg (DE), Green-house	6’904	14’224/12’112	4’049/4’171	GSM9220337 and GSM9220339
sid 2022	Leaf develop-mental zone	Heidelberg (DE), Green-house	7’437	15’201/13’354	4’112/4’250	GSM9220338 and GSM9220340
WT 2023-05-16	SAM & leaf primordia	Heidelberg (DE), Growth chamber	4’995	5’055/4’194	2’302/2’153	GSM9220341
WT 2023-05-17	SAM & leaf primordia	Heidelberg (DE), Growth chamber	2’674	4’798/3’938	2’230/2’085	GSM9220342
WT 2024-1	Leaf develop-mental zone	Bern (CH), Growth cham-ber	13’441	5’087/3’956	2’223/2’039	GSM9220343
WT 2024-2	Leaf develop-mental zone	Bern (CH), Growth cham-ber	11’546	6’304/5’366	2’720/2’626	GSM9220344
WT 2024-3	Leaf develop-mental zone	Bern (CH), Growth cham-ber	16’607	6’734/5’955	2’841/2’763	GSM9220345

Table S2. Necessary reported information to allow evaluation and repetition of a plant single cell/nucleus experiment, template from Grones et al. 2024

	Details	Experimental information
**Biological material**	Species	*Brachypodium distachyon*
	Accession	Bd21-3
	Genotype	WT and *sid/bdmute-1*
	Tissue type	Leaf developmental zones and vegetative shoot apex with leaf primordia
	Detailed growth conditions	Greenhouse or growth chamber, 18 h light: 6 h dark (day temperature 28°C, night temperature 22°C), PPFD 200-400 µmol m^{-2} s^{-1}, soil-grown (four parts Einheitserde CL ED73, 1 part Vermiculite), 2 days vernalization (in water, 4°C, dark)
	Harvest conditions	3-week-old plants, morning, 15 leaf developmental zones or 8-10 shoot apices
**Sample preparation**	Isolation protocol	Tissue was cut mechanically, incubated in water, digested using Cellulase “Onozuka RS” and Macerozyme R-10, and carefully pipetted up and down to release protoplasts
	Tissue dissection	Young, not yet unrolled leaves were pulled out from the enveloping older leaf and lowest 3-5 mm were cut off with a scalpel to harvest developmental zones. Shoot apices were dissected and harvested using a dissecting scope, forceps and a scalpel
	Fixation	Unfixed tissue
	Cell enrichment	Not applicable
	Total sample preparation time	4 to 4.5 h (from harvest to submission for 1-2 samples at a time, time includes counting of cells at the microscope and time needed to dilute to final concentration),<br>2.5 to 3 h (from digestion start to submission for 1-2 samples at a time)
	Estimated cell number loaded	12k cells (2021 datasets), 16.5k cells (2022 datasets), 18k cells (2023 and 2024 datasets)
	Instrument/Method/Kit	10x Genomics Chromium Single Cell 3’ v3.1
	Cell viability test	Fluorescein diacetate staining
**Libraries**	Library construction	GEM wells were prepared to encapsulate protoplasts in droplets, cDNA was extracted per protoplast and libraries were prepared according to the guidelines provided by 10x Genomics (single cell 3’ version 3.1)
	Amplification method	12 PCR cycles to amplify cDNA
	End bias	3’ end
**Sequence results**	Instrument/method	NextSeq 550 (2021 datasets, first sequencing run of the 2022 datasets, read length 100 bp),<br>NextSeq 2000 (second sequencing run of the 2022 datasets, 2023 datasets, read length 100 bp),<br>Illumina NovaSeq 6000 (2024 datasets, read length 150 bp)
	Library layout/paired-end	Paired-end
	N° sequenced reads<br>Reads/cell	~32-54k (2021 datasets),<br>~75-84k (2022 datasets),<br>~93-154k (2023 datasets),<br>~24k-29k (2024 datasets)

**Raw data**	Reference genome	https://phytozome-next.jgi.doe.gov/info/BdistachyonBd21_3_v1_2
	Annotation version	B. distachyon Bd21-3 v1.2
	Mapping method (incl. software, customized settings)	10x Genomics Cell Ranger v7.0.1
	Mapping efficiency	> 91% to > 97%
	Sequencing saturation	38-54% (2021 datasets),<br>42-45% (2022 datasets),<br>84-90% (2023 datasets),<br>54-61% (2024 datasets)
	Estimation of ambient RNA (Fraction reads in cell)	> 66% (2021 datasets),<br>> 71-77% (2022 datasets),<br>> 82-89% (2023 datasets),<br>> 75-85% (2024 datasets)
	Imputation method and settings	Not applicable
**Processed data**	N° captured cells (before filtering)	5'480 (WT 2021),<br>6'023 (sid/bdmute-1 2021),<br>10'023 (WT 2022),<br>11'438 (sid/bdmute-1 2022),<br>7'600 (WT 2023-05-16),<br>4'285 (WT 2023-05-17),<br>22'531 (WT 2024-1),<br>18'565 (WT 2024-2),<br>22'828 (WT 2024-3)
	N° high quality cells	69'687
	Filter criteria: % mitochondrial reads/cell	< 5%
	Filter criteria: % chloroplast reads/cell	< 10%
	Filter criteria: Minimum N° UMI/cell	> 1'250 and < 50'000 UMI;<br>> 500 and < 10'000 features
	N° total detected transcripts	> 91% (35'584 out of 39'068 genes)
	Doublet rate	6% (2021 datasets), 8% (2022 datasets), 10% (2023 and 2024 datasets)
	Replicate comparisons	Bulk RNA-seq protoplasted vs. non-protoplasted tissues (read length 75 bp)
	Batch correction method for merging (incl. reasoning for batch correction)	Semi-supervised integration with STACAS to correct for batch effects across libraries
	Additional processing	High ambient RNA filtering (SoupX), doublet removal (DoubletFinder)
**Validation**	Method of automatic annotation of clusters	Not applicable
	Method of manual annotation (markers, gene function info)	Marker genes (*B. distachyon*, orthologs to genes known from other species; see Table S3)
	Verification in planta (e.g. Number of markers used for validation)	13 marker genes verified with Hairpin Chain Reaction (HCR) RNA-fluorescence in situ hybridization,<br>4 marker genes verified with transcriptional reporter lines

**Data availability**	Analysis scripts & codes (GitHub)	Scripts available on Github: https://github.com/raissig-lindner-lab/Berg-et-al_2025_ScSeq
	Excel Tables DEG for each cluster	Supplementary information of the publication, Github: https://github.com/raissig-lindner-lab/Berg-et-al_2025_ScSeq
	Objects/count matrix in repository (which one, where?)	Single-cell data on GEO: GSE307277
	On-line tool/browser URL	https://shiny.ips.unibe.ch/
	Cell-level metadata table	Available in the R Script on Github: https://github.com/raissig-lindner-lab/Berg-et-al_2025_ScSeq
**Additional**	additional comments from the authors	Intronic reads were considered in Cell Ranger 7.0.1

Table S3. List of genes mentioned in the main text or figures of this paper.

Name	Bd21-3 accession	Bradi accession	Related gene in other species	Relevant literature
2cRR	BdiBd21-3.3G0655900	Bradi3g49440	N/A	This paper
BdAPL	BdiBd21-3.3G0071200	Bradi3g05500	*AtAPL*	(Bonke et al. 2003)
BdAGO10	BdiBd21-3.1G0499900	Bradi1g36907	*AtAGO10/PNH/ZLL*	(Lynn et al. 1999)
BdCLV1	BdiBd21-3.1G0402200	Bradi1g30160	*AtCLV1, HvCLV1* (HORVU.MOREX.r3.7HG0747230), *OsFON1, ZmTD1*	(Clark et al. 1993; Suzaki et al. 2004; Bommert et al. 2005; Demesa-Arevalo et al. 2025; Vardanega et al. 2025)
BdCRC	BdiBd21-3.1G0942300	Bradi1g69900	*HvCRC* (HORVU.MOREX.r3.4HG0396510)	(Bowman and Smyth 1999; Yamaguchi et al. 2004; Demesa-Arevalo et al. 2025)
BdCST1	BdiBd21-3.2G0326500	Bradi2g24850	*ZmCST1*	(Wang et al. 2019b)
BdEPF2-1	BdiBd21-3.5G0153600	Bradi5g12220	*AtEPF2, TaEPF2*	(Hara et al. 2009; Hunt and Gray 2009; Jangra et al. 2021)
BdEPF2-2	BdiBd21-3.5G0306100	Bradi5g23357	*AtEPF2, TaEPF1*	(Hara et al. 2009; Hunt and Gray 2009; Jangra et al. 2021)
BdERECTA	BdiBd21-3.1G0609800	Bradi1g46450	*AtERECTA*	(Shpak et al. 2005; Herrmann and Torii 2021; Chua and Lau 2024)
BdERL1	BdiBd21-3.1G0662500	Bradi1g49950	*AtERL1*	(Shpak et al. 2005; Herrmann and Torii 2021; Chua and Lau 2024)
BdFAMA	BdiBd21-3.2G0300000	Bradi2g22810	*AtFAMA, OsFAMA*	(Ohashi-Ito and Bergmann 2006; Liu et al. 2009; Wu et al. 2019; McKown et al. 2023)
BdFCP1	BdiBd21-3.5G0168500	Bradi5g13241	*OsFCP1, HvFCP1* (HORVU.MOREX.r3.2HG0174890), *ZmFCP1*	(Ohmori et al. 2013; Je et al. 2016; Vardanega et al. 2025)
BdFEA2	BdiBd21-3.2G0427700	Bradi2g34337	*ZmFEA2, AtCLV2*	(Je et al. 2018)
BdFEA3	BdiBd21-3.2G0010000	Bradi2g00920	*ZmFEA3, HvFEA3*	(Je et al. 2016; Demesa-Arevalo et al. 2025)
BdFEA4	BdiBd21-3.1G0571300	Bradi1g43900	*ZmFEA4*	(Pautler et al. 2015)
BdGELP1	BdiBd21-3.2G0049500	Bradi2g03807	*ZmGELP1*	(Sun et al. 2022)
BdGRAS32	BdiBd21-3.1G0657800	Bradi1g49630	*OsDLT/GRAS-32*	(Xie et al. 2019)
BdHDG2-like	BdiBd21-3.3G0192600	Bradi3g14500	*AtHDG2*	(Nakamura et al. 2006)
BdICE1	BdiBd21-3.4G0254800	Bradi4g17460	*AtICE1, OsICE1, ZmICEb*	(Kanaoka et al. 2008; Grimault et al. 2015; Raissig et al. 2016)
BdKN1	BdiBd21-3.1G0135700	Bradi1g10047	*ZmKN1*	(Smith et al. 1992)
BdKNAT1-like	BdiBd21-3.1G0773000	Bradi1g57607	*AtKNAT1*	(Lincoln et al. 1994)
BdLHCA6	BdiBd21-3.4G0439300	Bradi4g31257	*AtLHCA6*	(Jansson 1999)
BdMKK4/5-like1	BdiBd21-3.1G0616400	Bradi1g46880	*AtMKK4/5*	(Wang et al. 2007)
BdMKK4/5-like2	BdiBd21-3.3G0709500	Bradi3g53650	*AtMKK4/5*	(Wang et al. 2007)
BdMPK3-like	BdiBd21-3.1G0885800	Bradi1g65810	*AtMPK3*	(Wang et al. 2007)
BdMPK6-like	BdiBd21-3.1G0650300	Bradi1g49100	*AtMPK6*	(Wang et al. 2007)
BdMUTE	BdiBd21-3.1G0240400	Bradi1g18400	*AtMUTE, OsMUTE, ZmMUTE/BZU2*	(Pillitteri and Torii 2007; Raissig et al. 2017; Wang et al. 2019a; Wu et al. 2019; Spiegelhalder et al. 2024)
BdMYB60-like	BdiBd21-3.4G0234500	Bradi4g16290	*AtMYB60*	(Cominelli et al. 2005)
BdMYB88-like	BdiBd21-3.1G0268700	Bradi1g20586	*AtMYB88*	(Lai et al. 2005)
BdPAN1	BdiBd21-3.3G0526300	Bradi3g39910	*ZmPAN1*	(Cartwright et al. 2009; Zhang et al. 2022)
BdPAN2	BdiBd21-3.1G0783500	Bradi1g58260	*ZmPAN2*	(Zhang et al. 2012)
BdPDF1	BdiBd21-3.2G0082000	Bradi2g06300	*AtPDF1*	(Abe et al. 1999)
BdPDF2-like	BdiBd21-3.3G0204900	Bradi3g15327	*AtPDF2*	(Abe et al. 2003)
BdPHB-like	BdiBd21-3.1G0185500	Bradi1g13910	*AtPHB, AtPHV*	(McConnell et al. 2001; Emery et al. 2003)

Name	Bd21-3 accession	Bradi accession	Related gene in other species	Relevant literature
BdPIN1a	BdiBd21-3.1G0588300	Bradi1g45020	*HvPIN1a* (HORVU.MOREX.r3.7HG0666880)	(O'Connor et al. 2014; Fusi et al. 2024)
BdPIN1b	BdiBd21-3.3G0783600	Bradi3g59520	*HvPIN1/HvPIN1b* (HORVU.MOREX.r3.6HG0615550)	(O'Connor et al. 2014; Kirschner et al. 2018; Fusi et al. 2024)
BdPME53-like	BdiBd21-3.2G0255500	Bradi2g19420	*AtPME53*	(Wu et al. 2022)
BdSCAP1-like	BdiBd21-3.1G0206300	Bradi1g15420	*AtSCAP1*	(Negi et al. 2013)
BdSCRM2	BdiBd21-3.2G0762000	Bradi2g59497	*OsSCRM2*	(Raissig et al. 2016; Wu et al. 2019)
BdSDD1	BdiBd21-3.1G1009000	Bradi1g75550	*AtSDD1*	(Von Groll et al. 2002)
BdSERK1/2-like1	BdiBd21-3.5G0153700	Bradi5g12227	*AtSERK1/2*	(Meng et al. 2015)
BdSERK1/2-like2	BdiBd21-3.3G0620800	Bradi3g46747	*AtSERK1/2*	(Meng et al. 2015)
BdSERK1/2-like3	BdiBd21-3.3G0209300	Bradi3g15660	*AtSERK1/2*	(Meng et al. 2015)
BdSLAH2	BdiBd21-3.2G0203400	Bradi2g15500	*AtSLAH2*	(Maierhofer et al. 2014)
BdSLP1-like1	BdiBd21-3.3G0292100	Bradi3g21000	*SbSLP1*	(Kumar et al. 2020)
BdSLP1-like2	BdiBd21-3.3G0292000	Bradi3g20980	*SbSLP1*	(Kumar et al. 2020)
BdSLP1-like3	BdiBd21-3.3G0292700	Bradi3g21030	*SbSLP1*	(Kumar et al. 2020)
BdSLP1-like4	BdiBd21-3.5G0061500	Bradi5g04630	*SbSLP1*	(Kumar et al. 2020)
BdSLP1-like5	BdiBd21-3.1G0921800	Bradi1g68400	*SbSLP1*	(Kumar et al. 2020)
BdSLP1-like6	BdiBd21-3.1G0921700	Bradi1g68390	*SbSLP1*	(Kumar et al. 2020)
BdSLP1-like7	BdiBd21-3.3G0291900	Bradi3g20970	*SbSLP1*	(Kumar et al. 2020)
BdSLP1-like8	BdiBd21-3.3G0292200	Bradi3g21010	*SbSLP1*	(Kumar et al. 2020)
BdSLP1-like9	BdiBd21-3.1G0855400	Bradi1g63370	*SbSLP1*	(Kumar et al. 2020)
BdSPCH1	BdiBd21-3.1G0523400	Bradi1g38650	*AtSPCH, OsSPCH1*	(Pillitteri and Torii 2007; Raissig et al. 2016; Wu et al. 2019)
BdSPCH2	BdiBd21-3.3G0131200	Bradi3g09670	*AtSPCH, OsSPCH2*	(Pillitteri and Torii 2007; Liu et al. 2009; Raissig et al. 2016; Wu et al. 2019)
BdSPL10	BdiBd21-3.1G0421000	Bradi1g31390	*OsSPL10, ZmSPL14*	(Lan et al. 2019; Kong et al. 2024)
BdSPL5	BdiBd21-3.3G0074300	Bradi3g05720	*OsSPL5, ZmSPL10*	(Xie et al. 2006; Kong et al. 2024)
BdSTOMAGEN-1	BdiBd21-3.2G0749400	Bradi2g58540	*AtSTOMAGEN, TaSTOMAGEN-1*	(Sugano et al. 2010; Jangra et al. 2021)
BdSWEET13	BdiBd21-3.4G0103700	Bradi4g07570	*ZmSWEET13*	(Bezrutczyk et al. 2021)
BdTCP4-like	BdiBd21-3.2G0089600	Bradi2g06890	*AtTCP4*	(Palatnik et al. 2003)
BdTED4-like	BdiBd21-3.2G0231400	Bradi2g17540	*ZeTED4, AtTED4*	(Demura and Fukuda 1994; Endo et al. 2018)
BdTMM	BdiBd21-3.2G0561000	Bradi2g43940	*AtTMM*	(Geisler et al. 1998)
BdTMO6-like	BdiBd21-3.1G0348000	Bradi1g26570	*AtTMO6*	(Schlereth et al. 2010)
BdTSO1-like	BdiBd21-3.1G0741600	Bradi1g55710	*AtTSO1*	(Simmons et al. 2019)
BdVND-like1	BdiBd21-3.1G1023300	Bradi1g76732	*AtVNDs*	(Lehmann and Schneider 2025)
BdVND-like2	BdiBd21-3.5G0221500	Bradi5g16917	*AtVNDs*	(Lehmann and Schneider 2025)
BdWOX3B	BdiBd21-3.2G0477300	Bradi2g37650	*OsWOX3B, ZmWOX3A*	(Angeles-Shim et al. 2012; Li et al. 2012; Kong et al. 2021, 2024)
BdWOX4	BdiBd21-3.5G0316500	Bradi5g24080	*OsWOX4*	(Ohmori et al. 2013)
BdWOX9C-like1	BdiBd21-3.2G0587500	Bradi2g46055	*AtWOX9*	(Haecker et al. 2004)
BdXCP1-like	BdiBd21-3.2G0501500	Bradi2g39320	*AtXCP1*	(Funk et al. 2002)
BdYDA1	BdiBd21-3.5G0238000	Bradi5g18180	*AtYDA, HvYDA1*	(Bergmann et al. 2004; Abrash et al. 2018; Liu et al. 2022)
BdYDA2	BdiBd21-3.3G0680900	Bradi3g51380	*AtYDA, HvYDA2*	(Bergmann et al. 2004; Abrash et al. 2018; Liu et al. 2022)
DUF567	BdiBd21-3.4G0612300	Bradi4g44178	N/A	This paper
LRR kinase	BdiBd21-3.2G0614100	Bradi2g48000	N/A	This paper

Supplementary References for Table S3

Abe M, Katsumata H, Komeda Y, and Takahashi T. Regulation of shoot epidermal cell differentiation by a pair of homeodomain proteins in Arabidopsis. Development. 2003:**130**(4):635–643. https://doi.org/10.1242/dev.00292

Abe M, Takahashi T, and Komeda Y. Cloning and characterization of an L1 layer-specific gene in Arabidopsis thaliana. Plant Cell Physiol. 1999:**40**(6):571–580. https://doi.org/10.1093/oxfordjournals.pcp.a029579

Abrash E, Anleu Gil MX, Matos JL, and Bergmann DC. Conservation and divergence of YODA MAPKKK function in regulation of grass epidermal patterning. Development. 2018:**145**(14). https://doi.org/10.1242/dev.165860

Angeles-Shim RB, Asano K, Takashi T, Shim J, Kuroha T, Ayano M, and Ashikari M. A WUSCHEL-related homeobox 3B gene, depilous (dep), confers glabrousness of rice leaves and glumes. Rice . 2012:**5**(1):28. https://doi.org/10.1186/1939-8433-5-28

Bergmann DC, Lukowitz W, and Somerville CR. Stomatal development and pattern controlled by a MAPKK kinase. Science. 2004:**304**(5676):1494–1497.

Bezrutczyk M, Zöllner NR, Kruse CPS, Hartwig T, Lautwein T, Köhrer K, Frommer WB, and Kim J-Y. Evidence for phloem loading via the abaxial bundle sheath cells in maize leaves. Plant Cell. 2021:**33**(3):531–547. https://doi.org/10.1093/plcell/koaa055

Bommert P, Lunde C, Nardmann J, Vollbrecht E, Running M, Jackson D, Hake S, and Werr W. thick tassel dwarf1 encodes a putative maize ortholog of the Arabidopsis CLAVATA1 leucine-rich repeat receptor-like kinase. Development. 2005:**132**(6):1235–1245. https://doi.org/10.1242/dev.01671

Bonke M, Thitamadee S, Mähönen AP, Hauser M-T, and Helariutta Y. APL regulates vascular tissue identity in Arabidopsis. Nature. 2003:**426**(6963):181–186. https://doi.org/10.1038/nature02100

Bowman JL and Smyth DR. CRABS CLAW, a gene that regulates carpel and nectary development in Arabidopsis, encodes a novel protein with zinc finger and helix-loop-helix domains. Development. 1999:**126**(11):2387–2396. https://doi.org/10.1242/dev.126.11.2387

Cartwright HN, Humphries JA, and Smith LG. PAN1: a receptor-like protein that promotes polarization of an asymmetric cell division in maize. Science. 2009:**323**(5914):649–651. https://doi.org/10.1126/science.1161686

Chua LC and Lau OS. Stomatal development in the changing climate. Development. 2024:**151**(20):dev202681. https://doi.org/10.1242/dev.202681

Clark SE, Running MP, and Meyerowitz EM. CLAVATA1, a regulator of meristem and flower development in Arabidopsis. Development. 1993:**119**(2):397–418. https://doi.org/10.1242/dev.119.2.397

Cominelli E, Galbiati M, Vavasseur A, Conti L, Sala T, Vuylsteke M, Leonhardt N, Dellaporta SL, and Tonelli C. A guard-cell-specific MYB transcription factor regulates stomatal movements and plant drought tolerance. Curr Biol. 2005:**15**(13):1196–1200. https://doi.org/10.1016/j.cub.2005.05.048

Demesa-Arevalo E, Dörpholz H, Vardanega I, Maika JE, Pineda-Valentino I, Eggels S, Lautwein T, Köhrer K, Schnurbusch T, von Korff M, et al. Imputation integrates single-cell and spatial gene expression data to resolve transcriptional networks in barley shoot meristem development. bioRxiv. 2025:2025.05.09.653223. https://doi.org/10.1101/2025.05.09.653223

Demura T and Fukuda H. Novel vascular cell-specific genes whose expression is regulated temporally and spatially during vascular system development. Plant Cell. 1994:**6**(7):967–981. https://doi.org/10.1105/tpc.6.7.967

Emery JF, Floyd SK, Alvarez J, Eshed Y, Hawker NP, Izhaki A, Baum SF, and Bowman JL. Radial Patterning of Arabidopsis Shoots by Class III HD-ZIP and KANADI Genes. Current Biology. 2003:**13**(20):1768–1774. https://doi.org/10.1016/j.cub.2003.09.035

Endo S, Iwamoto K, and Fukuda H. Overexpression and cosuppression of xylem-related genes in an early xylem differentiation stage-specific manner by the AtTED4 promoter. Plant Biotechnol J. 2018:**16**(2):451–458. https://doi.org/10.1111/pbi.12784

Funk V, Kositsup B, Zhao C, and Beers EP. The Arabidopsis xylem peptidase XCP1 is a tracheary element vacuolar protein that may be a papain ortholog. Plant Physiol. 2002:**128**(1):84–94. https://doi.org/10.1104/pp.010514

Fusi R, Milner SG, Rosignoli S, Bovina R, De Jesus Vieira Teixeira C, Lou H, Atkinson BS, Borkar AN, York LM, Jones DH, et al. The auxin efflux carrier PIN1a regulates vascular patterning in cereal roots. New Phytol. 2024. https://doi.org/10.1111/nph.19777

Geisler M, Yang M, and Sack FD. Divergent regulation of stomatal initiation and patterning in organ and suborgan regions of the Arabidopsis mutants too many mouths and four lips. Planta. 1998:**205**(4):522–530.

Grimault A, Gendrot G, Chamot S, Widiez T, Rabillé H, Gérentes M-F, Creff A, Thévenin J, Dubreucq B, Ingram GC, et al. ZmZHOUPI, an endosperm-specific basic helix-loop-helix transcription factor involved in maize seed development. Plant J. 2015:**84**(3):574–586. https://doi.org/10.1111/tpj.13024

Haecker A, Gross-Hardt R, Geiges B, Sarkar A, Breuninger H, Herrmann M, and Laux T. Expression dynamics of WOX genes mark cell fate decisions during early embryonic patterning in Arabidopsis thaliana. Development. 2004:**131**(3):657–668. https://doi.org/10.1242/dev.00963

Hara K, Yokoo T, Kajita R, Onishi T, Yahata S, Peterson KM, Torii KU, and Kakimoto T. Epidermal cell density is autoregulated via a secretory peptide, EPIDERMAL PATTERNING FACTOR 2 in Arabidopsis leaves. Plant Cell Physiol. 2009:**50**(6):1019–1031.

Herrmann A and Torii KU. Shouting out loud: signaling modules in the regulation of stomatal development. Plant Physiol. 2021:**185**(3):765–780. https://doi.org/10.1093/plphys/kiaa061

Hunt L and Gray JE. The signaling peptide EPF2 controls asymmetric cell divisions during stomatal development. Curr Biol. 2009:**19**(10):864–869.

Jangra R, Brunetti SC, Wang X, Kaushik P, Gulick PJ, Foroud NA, Wang S, and Lee JS. Duplicated antagonistic EPF peptides optimize grass stomatal initiation. Development. 2021:**148**(16):dev199780. https://doi.org/10.1242/dev.199780

Jansson S. A guide to the Lhc genes and their relatives in Arabidopsis/IT>. Trends Plant Sci. 1999:**4**(6):236–240. https://doi.org/10.1016/s1360-1385(99)01419-3

Je BI, Gruel J, Lee YK, Bommert P, Arevalo ED, Eveland AL, Wu Q, Goldshmidt A, Meeley R, Bartlett M, et al. Signaling from maize organ primordia via FASCIATED EAR3 regulates stem cell proliferation and yield traits. Nat Genet. 2016:**48**(7):785–791. https://doi.org/10.1038/ng.3567

Je BI, Xu F, Wu Q, Liu L, Meeley R, Gallagher JP, Corcilius L, Payne RJ, Bartlett ME, and Jackson D. The CLAVATA receptor FASCIATED EAR2 responds to distinct CLE peptides by signaling through two downstream effectors. Elife. 2018:**7**. https://doi.org/10.7554/eLife.35673

Kanaoka MM, Pillitteri LJ, Fujii H, Yoshida Y, Bogenschutz NL, Takabayashi J, Zhu J-K, and Torii KU. SCREAM/ICE1 and SCREAM2 specify three cell-state transitional steps leading to arabidopsis stomatal differentiation. Plant Cell. 2008:**20**(7):1775–1785.

Kirschner GK, Stahl Y, Imani J, von Korff M, and Simon R. Fluorescent reporter lines for auxin and cytokinin signalling in barley (Hordeum vulgare). PLoS One. 2018:**13**(4):e0196086. https://doi.org/10.1371/journal.pone.0196086

Kong D, Jing Y, Duan Y, He M, Ding H, Li H, Zhong Z, Zheng Z, Fan X, Pan X, et al. ZmSPL10, ZmSPL14 and ZmSPL26 act together to promote stigmatic papilla formation in maize through regulating auxin signaling and ZmWOX3A expression. New Phytol. 2024:**243**(5):1870–1886. https://doi.org/10.1111/nph.19961

Kong D, Pan X, Jing Y, Zhao Y, Duan Y, Yang J, Wang B, Liu Y, Shen R, Cao Y, et al. ZmSPL10/14/26 are required for epidermal hair cell fate specification on maize leaf. New Phytol. 2021:**230**(4):1533–1549. https://doi.org/10.1111/nph.17293

Kumar S, Adiram-Filiba N, Blum S, Sanchez-Lopez JA, Tzfadia O, Omid A, Volpin H, Heifetz Y, Goobes G, and Elbaum R. Siliplant1 protein precipitates silica in sorghum silica cells. J Exp Bot. 2020:**71**(21):6830–6843. https://doi.org/10.1093/jxb/eraa258

Lai LB, Nadeau JA, Lucas J, Lee E-K, Nakagawa T, Zhao L, Geisler M, and Sack FD. The Arabidopsis R2R3 MYB proteins FOUR LIPS and MYB88 restrict divisions late in the stomatal cell lineage. Plant Cell. 2005:**17**(10):2754–2767. https://doi.org/10.1105/tpc.105.034116

Lan T, Zheng Y, Su Z, Yu S, Song H, Zheng X, Lin G, and Wu W. OsSPL10, a SBP-box gene, plays a dual role in salt tolerance and trichome formation in rice (Oryza sativa L.). G3 (Bethesda). 2019:**9**(12):4107–4114. https://doi.org/10.1534/g3.119.400700

Lehmann U and Schneider R. The role of VND transcription factors in xylem vessel development and secondary wall formation. New Phytol. 2025:**247**(5):2034–2041. https://doi.org/10.1111/nph.70327

Li J, Yuan Y, Lu Z, Yang L, Gao R, Lu J, Li J, and Xiong G. Glabrous Rice 1, encoding a homeodomain protein, regulates trichome development in rice. Rice (N Y). 2012:**5**(1):32. https://doi.org/10.1186/1939-8433-5-32

Lincoln C, Long J, Yamaguchi J, Serikawa K, and Hake S. A knotted1-like homeobox gene in Arabidopsis is expressed in the vegetative meristem and dramatically alters leaf morphology when overexpressed in transgenic plants. Plant Cell. 1994:**6**(12):1859–1876. https://doi.org/10.1105/tpc.6.12.1859

Liu L, Jose SB, Campoli C, Bayer MM, Sánchez-Diaz MA, McAllister T, Zhou Y, Eskan M, Milne L, Schreiber M, et al. Conserved signalling components coordinate epidermal patterning and cuticle deposition in barley. Nat Commun. 2022:**13**(1):6050. https://doi.org/10.1038/s41467-022-33300-1

Liu T, Ohashi-Ito K, and Bergmann DC. Orthologs of Arabidopsis thaliana stomatal bHLH genes and regulation of stomatal development in grasses. Development. 2009:**136**(13):2265–2276.

Lynn K, Fernandez A, Aida M, Sedbrook J, Tasaka M, Masson P, and Barton MK. The PINHEAD/ZWILLE gene acts pleiotropically in Arabidopsis development and has overlapping functions with the ARGONAUTE1 gene. Development. 1999:**126**(3):469–481. https://doi.org/10.1242/dev.126.3.469

Maierhofer T, Lind C, Hüttl S, Scherzer S, Papenfuß M, Simon J, Al-Rasheid KAS, Ache P, Rennenberg H, Hedrich R, et al. A single-pore residue renders the Arabidopsis root anion channel SLAH2 highly nitrate selective. Plant Cell. 2014:**26**(6):2554–2567. https://doi.org/10.1105/tpc.114.125849

McConnell JR, Emery J, Eshed Y, Bao N, Bowman J, and Barton MK. Role of PHABULOSA and PHAVOLUTA in determining radial patterning in shoots. Nature. 2001:**411**(6838):709–713. https://doi.org/10.1038/35079635

McKown KH, Anleu Gil MX, Mair A, Xu S-L, Raissig MT, and Bergmann DC. Expanded roles and divergent regulation of FAMA in Brachypodium and Arabidopsis stomatal development. Plant Cell. 2023:**35**(2):756–775. https://doi.org/10.1093/plcell/koac341

Meng X, Chen X, Mang H, Liu C, Yu X, Gao X, Torii KU, He P, and Shan L. Differential Function of Arabidopsis SERK Family Receptor-like Kinases in Stomatal Patterning. Curr Biol. 2015:**25**(18):2361–2372. https://doi.org/10.1016/j.cub.2015.07.068

Nakamura M, Katsumata H, Abe M, Yabe N, Komeda Y, Yamamoto KT, and Takahashi T. Characterization of the class IV homeodomain-Leucine Zipper gene family in Arabidopsis. Plant Physiol. 2006:**141**(4):1363–1375. https://doi.org/10.1104/pp.106.077388

Negi J, Moriwaki K, Konishi M, Yokoyama R, Nakano T, Kusumi K, Hashimoto-Sugimoto M, Schroeder JI, Nishitani K, Yanagisawa S, et al. A Dof transcription factor, SCAP1, is essential for the development of functional stomata in Arabidopsis. Curr Biol. 2013:**23**(6):479–484. https://doi.org/10.1016/j.cub.2013.02.001

O'Connor DL, Runions A, Sluis A, Bragg J, Vogel JP, Prusinkiewicz P, and Hake S. A division in PIN-mediated auxin patterning during organ initiation in grasses. PLoS Comput Biol. 2014:**10**(1):e1003447. https://doi.org/10.1371/journal.pcbi.1003447

Ohashi-Ito K and Bergmann DC. Arabidopsis FAMA controls the final proliferation/differentiation switch during stomatal development. Plant Cell. 2006:**18**(10):2493–2505.

Ohmori Y, Tanaka W, Kojima M, Sakakibara H, and Hirano H-Y. WUSCHEL-RELATED HOMEOBOX4 is involved in meristem maintenance and is negatively regulated by the CLE gene FCP1 in rice. Plant Cell. 2013:**25**(1):229–241. https://doi.org/10.1105/tpc.112.103432

Palatnik JF, Allen E, Wu X, Schommer C, Schwab R, Carrington JC, and Weigel D. Control of leaf morphogenesis by microRNAs. Nature. 2003:**425**(6955):257–263. https://doi.org/10.1038/nature01958

Pautler M, Eveland AL, LaRue T, Yang F, Weeks R, Lunde C, Je BI, Meeley R, Komatsu M, Vollbrecht E, et al. FASCIATED EAR4 encodes a bZIP transcription factor that regulates shoot meristem size in maize. Plant Cell. 2015:**27**(1):104–120. https://doi.org/10.1105/tpc.114.132506

Pillitteri LJ and Torii KU. Breaking the silence: three bHLH proteins direct cell-fate decisions during stomatal development. BioEssays. 2007:**29**(9):861–870. https://doi.org/10.1002/bies.20625

Raissig MT, Abrash E, Bettadapur A, Vogel JP, and Bergmann DC. Grasses use an alternatively wired bHLH transcription factor network to establish stomatal identity. Proc Natl Acad Sci U S A. 2016:**113**(29):8326–8331. https://doi.org/10.1073/pnas.1606728113

Raissig MT, Matos JL, Anleu Gil MX, Kornfeld A, Bettadapur A, Abrash E, Allison HR, Badgley G, Vogel JP, Berry JA, et al. Mobile MUTE specifies subsidiary cells to build physiologically improved grass stomata. Science. 2017:**355**(6330):1215–1218. https://doi.org/10.1126/science.aal3254

Schlereth A, Möller B, Liu W, Kientz M, Flipse J, Rademacher EH, Schmid M, Jürgens G, and Weijers D. MONOPTEROS controls embryonic root initiation by regulating a mobile transcription factor. Nature. 2010:**464**(7290):913–916. https://doi.org/10.1038/nature08836

Shpak ED, McAbee JM, Pillitteri LJ, and Torii KU. Stomatal patterning and differentiation by synergistic interactions of receptor kinases. Science. 2005:**309**(5732):290–293.

Simmons AR, Davies KA, Wang W, Liu Z, and Bergmann DC. SOL1 and SOL2 regulate fate transition and cell divisions in the Arabidopsis stomatal lineage. Development. 2019:**146**(3):dev171066. https://doi.org/10.1242/dev.171066

Smith LG, Greene B, Veit B, and Hake S. A dominant mutation in the maize homeobox gene, Knotted-1, causes its ectopic expression in leaf cells with altered fates. Development. 1992:**116**(1):21–30. https://doi.org/10.1242/dev.116.1.21

Spiegelhalder RP, Berg LS, Nunes TDG, Dörr M, Jesenofsky B, Lindner H, and Raissig MT. Dual role of BdMUTE during stomatal development in the model grass Brachypodium distachyon. Development. 2024:**151**(20):dev.203011. https://doi.org/10.1242/dev.203011

Sugano SS, Shimada T, Imai Y, Okawa K, Tamai A, Mori M, and Hara-Nishimura I. Stomagen positively regulates stomatal density in Arabidopsis. Nature. 2010:**463**(7278):241–244. https://doi.org/10.1038/nature08682

Sun G, Xia M, Li J, Ma W, Li Q, Xie J, Bai S, Fang S, Sun T, Feng X, et al. The maize single-nucleus transcriptome comprehensively describes signaling networks governing movement and development of grass stomata. Plant Cell. 2022. https://doi.org/10.1093/plcell/koac047

Suzaki T, Sato M, Ashikari M, Miyoshi M, Nagato Y, and Hirano H-Y. The gene FLORAL ORGAN NUMBER1 regulates floral meristem size in rice and encodes a leucine-rich repeat receptor kinase orthologous to Arabidopsis CLAVATA1. Development. 2004:**131**(22):5649–5657. https://doi.org/10.1242/dev.01441

Vardanega I, Maika JE, Demesa-Arevalo E, Lan T, Kirschner GK, Imani J, Acosta IF, Makowska K, Hensel G, Ranaweera T, et al. CLAVATA signalling shapes barley inflorescence by controlling activity and determinacy of shoot meristem and rachilla. Nat Commun. 2025:**16**(1):1–14. https://doi.org/10.1038/s41467-025-59330-z

Von Groll U, Berger D, and Altmann T. The subtilisin-like serine protease SDD1 mediates cell-to-cell signaling during Arabidopsis stomatal development. Plant Cell. 2002:**14**(7):1527–1539. https://doi.org/10.1105/tpc.001016

Wang H, Guo S, Qiao X, Guo J, Li Z, Zhou Y, Bai S, Gao Z, Wang D, Wang P, et al. BZU2/ZmMUTE controls symmetrical division of guard mother cell and specifies neighbor cell fate in maize. PLoS Genet. 2019a:**15**(8):e1008377. https://doi.org/10.1371/journal.pgen.1008377

Wang H, Ngwenyama N, Liu Y, Walker JC, and Zhang S. Stomatal development and patterning are regulated by environmentally responsive mitogen-activated protein kinases in Arabidopsis. Plant Cell. 2007:**19**(1):63–73. https://doi.org/10.1105/tpc.106.048298

Wang H, Yan S, Xin H, Huang W, Zhang H, Teng S, Yu Y-C, Fernie AR, Lu X, Li P, et al. A Subsidiary Cell-Localized Glucose Transporter Promotes Stomatal Conductance and Photosynthesis. Plant Cell. 2019b:**31**(6):1328–1343. https://doi.org/10.1105/tpc.18.00736

Wu H-C, Yu S-Y, Wang Y-D, and Jinn T-L. Guard Cell-Specific Pectin METHYLESTERASE53 Is Required for Abscisic Acid-Mediated Stomatal Function and Heat Response in Arabidopsis. Front Plant Sci. 2022:**13**:836151. https://doi.org/10.3389/fpls.2022.836151

Wu Z, Chen L, Yu Q, Zhou W, Gou X, Li J, and Hou S. Multiple transcriptional factors control stomata development in rice. New Phytol. 2019:**223**(1):220–232. https://doi.org/10.1111/nph.15766

Xie J, Liao H, Wang X, Zhang X, Ni J, Li Y, Tian W, and Sang X. DLT/OsGRAS-32, regulating leaf width and thickness by controlling cell number in Oryza sativa. Mol Breed. 2019:**39**(7):104. https://doi.org/10.1007/s11032-019-1003-6

Xie K, Wu C, and Xiong L. Genomic organization, differential expression, and interaction of SQUAMOSA promoter-binding-like transcription factors and microRNA156 in rice. Plant Physiol. 2006:**142**(1):280–293. https://doi.org/10.1104/pp.106.084475

Yamaguchi T, Nagasawa N, Kawasaki S, Matsuoka M, Nagato Y, and Hirano H-Y. The YABBY gene DROOPING LEAF regulates carpel specification and midrib development in Oryza sativa. Plant Cell. 2004:**16**(2):500–509. https://doi.org/10.1105/tpc.018044

Zhang D, Spiegelhalder RP, Abrash EB, Nunes TDG, Hidalgo I, Anleu Gil MX, Jesenofsky B, Lindner H, Bergmann DC, and Raissig MT. Opposite polarity programs regulate asymmetric subsidiary cell divisions in grasses. eLife. 2022:**11**:e79913. https://doi.org/10.7554/eLife.79913

Zhang X, Facette M, Humphries JA, Shen Z, Park Y, Sutimantanapi D, Sylvester AW, Briggs SP, and Smith LG. Identification of PAN2 by Quantitative Proteomics as a Leucine-Rich Repeat–Receptor-Like Kinase Acting Upstream of PAN1 to Polarize Cell Division in Maize. Plant Cell. 2012:**24**(11):4577–4589. https://doi.org/10.1105/tpc.112.104125

Table S4. Seurat marker genes for all clusters

see separate file "TableS4.xlsx"

Table S5. Differentially expressed genes in non-protoplasted vs. protoplasted bulk RNA-seq

see separate file "TableS5.xlsx"

Table S6. Borda-ranked transcription factors per stomatal cluster (Mini-Ex analysis)

see separate file "TableS6.xlsx"

Table S7. Targetome of BdMUTE and BdFAMA in dividing GMC cluster

see separate file "TableS7.xlsx"

Table S8. Details on statistical analysis

see separate file "TableS8.xlsx"

Table S9. Key resources table

see separate file "TableS9.xlsx"
